# The Small Protein YmoA Controls the Csr System and Adjusts Expression of Virulence-Relevant Traits of *Yersinia pseudotuberculosis*

**DOI:** 10.3389/fmicb.2021.706934

**Published:** 2021-08-03

**Authors:** Katja Böhme, Ann Kathrin Heroven, Stephanie Lobedann, Yuzhu Guo, Anne-Sophie Stolle, Petra Dersch

**Affiliations:** ^1^Department of Molecular Infection Biology, Helmholtz Centre for Infection Research, Braunschweig, Germany; ^2^Institute of Infectiology, Center for Molecular Biology of Inflammation (ZMBE), Medical Faculty Münster, University of Münster, Münster, Germany

**Keywords:** *Yersinia*, regulatory RNA, gene regulation, virulence, CsrA, YmoA, Hha

## Abstract

Virulence gene expression of *Yersinia pseudotuberculosis* changes during the different stages of infection and this is tightly controlled by environmental cues. In this study, we show that the small protein YmoA, a member of the Hha family, is part of this process. It controls temperature- and nutrient-dependent early and later stage virulence genes in an opposing manner and co-regulates bacterial stress responses and metabolic functions. Our analysis further revealed that YmoA exerts this function by modulating the global post-transcriptional regulatory Csr system. YmoA pre-dominantly enhances the stability of the regulatory RNA CsrC. This involves a stabilizing stem-loop structure within the 5′-region of CsrC. YmoA-mediated CsrC stabilization depends on H-NS, but not on the RNA chaperone Hfq. YmoA-promoted reprogramming of the Csr system has severe consequences for the cell: we found that a mutant deficient of *ymoA* is strongly reduced in its ability to enter host cells and to disseminate to the Peyer’s patches, mesenteric lymph nodes, liver and spleen in mice. We propose a model in which YmoA controls transition from the initial colonization phase in the intestine toward the host defense phase important for the long-term establishment of the infection in underlying tissues.

## Introduction

Enteropathogenic yersiniae, *Yersinia enterocolitica* and *Yersinia pseudotuberculosis* are fecal-oral pathogens that can cause food-borne infections in animals and humans with symptoms ranging from self-limiting enteritis, mesenterial lymphadenitis to autoimmune responses (Bottone, 1997; Koornhof et al., 1999; Galindo et al., 2011). Both *Yersinia* species initiate infection after oral uptake in the gastrointestinal tract by tight adhesion to the mucosal surface of the intestine, which is followed by rapid internalization and translocation through M-cells of the intestinal epithelium (Pepe and Miller, 1993; Marra and Isberg, 1997; Koornhof et al., 1999). Migration through M-cells leads to the accumulation of the bacteria in underlying lymphatic tissues (Peyer’s patches) and their dissemination to mesenteric lymph nodes, liver and spleen (Barnes et al., 2006; Trülzsch et al., 2007).

**FIGURE 1:**
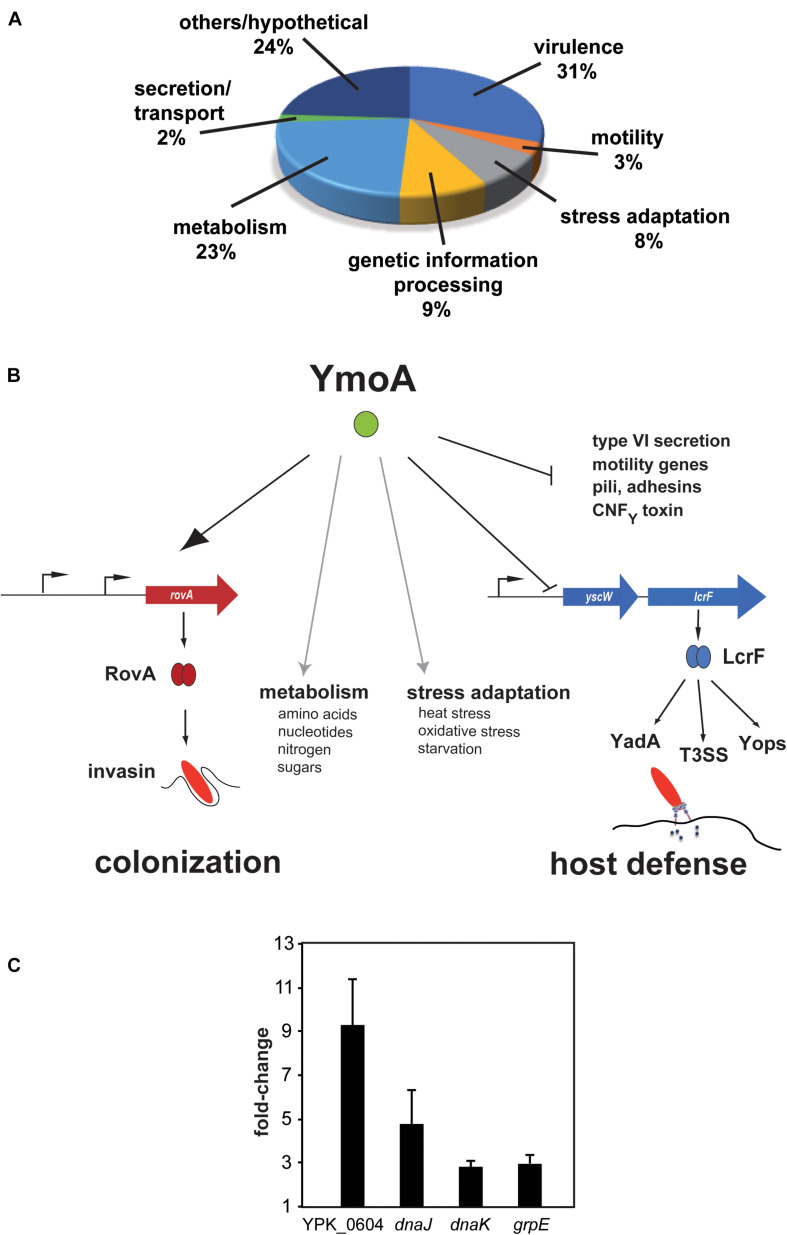
Influence of YmoA on the global gene expression in *Y. pseudotuberculosis.* **(A)** Proportion of gene classes up- or downregulated by YmoA. Genes showing overall fold-changes ≥ 2.0 are included in the displayed diagram of gene classes. **(B)** Schematic scheme of the global influence of YmoA on virulence-relevant genes. YmoA activates the synthesis of the early-phase virulence RovA, leading to expression of colonization factors such as invasin. In contrast, later-stage virulence factors, e.g., YadA, Yops, or T3SS, which are essential to combat triggered immune responses, are silenced by YmoA through repression of the transcriptional activator LcrF. Moreover, YmoA is implicated in many other virulence-relevant metabolic and stress adaptation processes. This control network allows switching of virulence gene expression by variations of YmoA levels. The dark arrow displays activation of the gene expression or protein synthesis; T represents repression or inactivation. Gray arrows indicate control of metabolic and stress adaptation processes by YmoA. **(C)** RNA of early stationary cultures of strains YPIII and YP50 (Δ*ymoA*) in LB medium at 25°C was prepared and six independent samples were pooled. qRT-PCR was carried out in triplicates. Gene expression levels were normalized to levels of the *sopB* transcript and are given as relative values Δ*ymoA*/wild-type. Mean ± SD of three independent experiments are displayed.

Upon ingestion, *Yersinia* encounters changing growth conditions including elevated temperature as well as variations of nutrients and ions within the intestinal tract. *Yersinia* senses changes of environmental parameters to determine its localization and adapt expression of pathogenicity factors and survival strategies accordingly (Marceau, 2005; Heroven and Dersch, 2014; Chen et al., 2016). Some of the most important virulence properties are encoded on the virulence plasmid pYV (pIB1). They are predominantly expressed at 37°C upon contact with host immune cells after the bacteria entered the gut-associated lymphatic tissues (Straley and Perry, 1995; Kusmierek et al., 2019; Volk et al., 2019; Schneiders et al., 2021). Among them are the adhesion factor YadA, the structural components of a functional type three secretion system (T3SS) and the antiphagocytic *Yersinia* outer proteins (Yops) - the effectors which are secreted and translocated by the T3S machinery (Cornelis, 2006; Bliska et al., 2013). Expression of these virulence genes is activated by the AraC-type transcriptional regulator LcrF (VirF) (Lambert de Rouvroit et al., 1992; Hoe and Goguen, 1993; Böhme et al., 2012; Schwiesow et al., 2015) (Figure 1).

Other virulence factors, including smooth lipopolysaccharides, flagellar motility, iron uptake and storage, enterotoxin (Yst) and the cell internalization factor invasin are predominantly expressed at moderate temperatures during stationary phase, conditions, which are more typical for the environmental life-style of the bacteria (Straley and Perry, 1995; Marceau, 2005; Heroven and Dersch, 2014; Chen et al., 2016). This class of virulence factors appears to be required for the initial stages of infection and seems to assure rapid and efficient colonization and penetration of the intestinal epithelial layer shortly after infection. The molecular mechanisms, regulating the expression of the virulence genes during the different stages of the infection are still far from being understood. However, over the last years it became evident that a complex regulatory network including the carbon storage regulator (Csr) system plays a crucial role in the control of virulence-associated traits initiating intestinal colonization and dissemination into deeper tissues (Heroven et al., 2008, 2012a; Nuss et al., 2014, 2017a, b; Kusmierek and Dersch, 2017; Kusmierek et al., 2019).

The Csr system is a global regulatory system that consists of (i) the dimeric RNA-binding protein CsrA, that binds to and thus regulates translation and stability of target mRNAs, and (ii) the untranslated regulatory Csr-RNAs CsrB and CsrC that antagonize CsrA function (Heroven et al., 2012a; Kusmierek and Dersch, 2017; Romeo and Babitzke, 2018; Pourciau et al., 2020). Both CsrB and CsrC bind multiple CsrA dimers. This sequestration of CsrA reduces the pool of free CsrA which is able to interact with other target mRNAs without changing the overall level of CsrA. The Csr system was first identified to control the expression of the virulence regulator RovA of *Y. pseudotuberculosis* (Heroven et al., 2008). RovA, a member of the SlyA/Hor family of DNA-binding proteins, acts as an activator of the primary cell internalization factor invasin, the PsaA adhesin (pH6 antigen) and many other factors contributing to *Yersinia* virulence in the early stages of the infection (Nagel et al., 2001; Cathelyn et al., 2006, 2007; Yang et al., 2010). CsrA-mediated influence on *rovA* expression occurs through indirect control of the LysR-type regulator RovM (Heroven and Dersch, 2006; Heroven et al., 2008). Further analysis revealed a complex control system in which the regulatory RNAs CsrC and CsrB are regulated by the global cAMP-binding repressor protein (Crp) and the two-component system BarA/UvrY or PhoQ/PhoP in response to nutrients/metabolites and ions (Heroven et al., 2008, 2012b; Bücker et al., 2014; Nuss et al., 2014).

Virulence-promoting processes are activated by the CsrABC-RovM-RovA cascade in the very early colonization stages of the infection. Once bacteria have reached the lymphatic tissues these virulence traits are no longer needed and hence are downregulated during ongoing infection stages. In this phase, virulence plasmid-encoded LcrF-induced defensive pathogenicity factors are upregulated to protect the pathogen against the attack of the host immune system. How reprogramming of virulence gene expression is controlled in the course of the infection is still unknown, however, it is emerging that the small protein YmoA (*Yersinia* modulator A; 9 kDa, 67 aa) participates in this process. YmoA shares extensive homology with the *Escherichia coli* Hha protein, and belongs to a group of low-molecular-weight proteins involved in the regulation of bacterial pathogenicity and virulence-associated physiological traits (Cornelis et al., 1991; de la Cruz et al., 1992; Mikulskis and Cornelis, 1994; Madrid et al., 2002, 2007; Lawal et al., 2013). YmoA was first identified as a thermosensitive repressor of *lcrF*, encoding the main transcriptional activator of the pYV-encoded later-stage virulence genes, and the *Y. enterocolitica* enterotoxin gene *yst* (Cornelis et al., 1991; de la Cruz et al., 1992; Cornelis, 1993; Mikulskis et al., 1994; Jackson et al., 2004; Böhme et al., 2012). Thermal control is mediated by rapid degradation of YmoA by Lon and Clp proteases at a host temperature of 37°C (Jackson et al., 2004; Böhme et al., 2012). Later on, several studies demonstrated that YmoA interacts with a small portion of the nucleoid-structuring and global regulatory protein H-NS, and this generates YmoA/H-NS heteromeric complexes with somewhat different target specificity (Madrid et al., 2002; Nieto et al., 2002; Ellison and Miller, 2006). This suggests that YmoA has a more global role and evolved to fine-tune the activity of H-NS to adapt expression of virulence-associated traits in response to temperature. More recently, is has been reported that YmoA/Hha together with YmoB/TomB(YbaJ) bears the characteristics of a bonafide type II toxin-antitoxin (TA) system, which is involved in biofilm and persister cell formation in *E. coli* and in persistence and programmed cell death in *Salmonella enterica* serovar Typhimurium (García-Contreras et al., 2008; Marimon et al., 2016).

In this study, we demonstrate that YmoA plays a key role in the control *Yersinia* pathogenesis. YmoA is part of a complex regulatory network and controls more than 289 genes, many of them implicated in virulence. In addition to its inhibitory effect on antiphagocytic T3SS-associated virulence factors, YmoA was found to promote the induction of the CsrABC-RovM-RovA regulatory cascade, which is important for the expression of early-stage virulence genes through control of CsrB and CsrC RNA levels. We postulate that YmoA in *Y. pseudotuberculosis* plays a crucial role switching expression from RovA-activated early-stage virulence genes toward LcrF-induced virulence genes important for host defense.

## Materials and Methods

### Cell Culture, Media and Growth Conditions

Overnight cultures of *E. coli* were routinely grown at 37°C, *Yersinia* strains were grown at 25°C or 37°C in LB (Luria Bertani) broth if not indicated otherwise. The antibiotics used for bacterial selection were as follows: ampicillin 100 μg ml^–1^, chloramphenicol 30 μg ml^–1^, tetracycline 5 μg ml^–1^, kanamycin 50 μg ml^–1^, and gentamicin 50 μg ml^–1^. HEp-2 cells were cultured in RPMI 1640 media (Sigma) supplemented with 5% new-born calf serum (Invitrogen) and 2 mM glutamine at 37°C in the presence of 5% CO_2_.

### Plasmid Constructions

All DNA manipulations, restriction digestions, ligations and transformations were performed using standard genetic and molecular techniques (Miller, 1992; Sambrook, 2001). Plasmid DNA was purified using a Qiagen kit. Restriction and DNA-modifying enzymes were obtained from Roche, Promega or New England Biolabs. The oligonucleotides used for amplification by PCR, sequencing and primer extension were purchased from Metabion (Martinsried, Germany). PCR reactions were performed routinely in a 100 μl mix for 25 cycles using *Taq* polymerase or Phusion High-Fidelity DNA polymerase (New England Biolabs) according to the manufacturer’s instructions. PCR products were purified with the QIAquick PCR purification kit (Qiagen, Germany) before and after digestion of the amplification product. Sequencing reactions were performed by Agowa (Berlin, Germany) or GATC (Konstanz, Germany).

Plasmids used in this study are listed in Supplementary Table 1 and primers for plasmid generation are listed in Supplementary Table 2. Plasmids pAKH106, pAKH107, pKB20, pAKH76 and pAKH104 carry PCR generated fragments harboring *csrC* promoter regions from nucleotide −355 (primer 3) to nucleotide +39 (primer 5), +61 (primer 6), +71 (primer 7), +81 (primer 8) and +254 (primer 9), respectively. The fragments were digested with *Eco*RI and *Sal*I and cloned into the corresponding sites of pHT124. The *hfq*^+^ plasmids pAKH115 and pAKH119 were constructed by insertion of a PCR fragment amplified with primer 10 and primer 11 into the vector pACYC184 and pHSG575 digested with *Sal*I and *Bam*HI.

To generate pKB4, the *ymoA* gene was amplified from chromosomal DNA of YPIII using primer 1 and 2 and inserted into the *Sal*I/*Bam*HI sites of pHSG575. To generate pKB17, a *csrC* fragment harboring an internal deletion from nucleotide +24 to +57 was amplified using primers 3 and 12 and cloned into the *Eco*RI/*Sal*I sites of pHT124. To express the *csrC* gene under the control of the *tet* promoter, *csrC* was amplified with primers 13 (including the *tet* promoter sequence) and 14. The resulting fragment was cloned into the *Sal*I/*Bam*HI sites of vector pHSG575, generating pKB47. The *csrC* gene, including the 5′-regulatory region, was amplified with primers 15 and 16 and inserted into the *Bam*HI/*Sal*I site of pHSG576, resulting in pKB59. Subsequently, the region from position +24 to +57 within the *csrC* gene was deleted by three-step-PCR. First, the upstream region was amplified by primers 15 and 17 (encoding the deleted region), and the downstream region was amplified with primer 18 (encoding the deleted region) and primer 16. Next, up- and downstream fragments were mixed with primers 15 and 16 to generate the *csrC* deletion fragment, which was inserted into the *Sal*I/*Bam*HI sites resulting in pKB49. The P*_*tet*_*:*lacZ* containing vector pTT1 was constructed by the insertion of a DNA fragment amplified with primers 23 and 24 into the *Pst*I/*Eco*RV sites of pTS03. The sequence and the correct orientation of all cloned fragments were proven by DNA sequencing.

### Construction of the *Y. pseudotuberculosis* Deletion Mutants

The *Y. pseudotuberculosis* mutant strains were constructed with the RED recombinase system as described previously (Datsenko and Wanner, 2000; Derbise et al., 2003) and are all derived from wild-type strain YPIII. First, a kanamycin cassette was amplified by PCR with primers homologous to the resistance gene encoded on pKD4 followed by homologous sequences of adjacent regions of the target gene (for primer see Supplementary Table 3). The PCR fragment was transformed into *Y. pseudotuberculosis* YPIII pKD46. Chromosomal integration of the fragment was selected by plating on LB supplemented with kanamycin. Subsequently, mutant derivatives were cured of the temperature-sensitive plasmid pKD46 by cultivation at 37°C. To remove the resistance gene at its FLP recognition sites the mutants were transformed with the helper plasmid pCP20 encoding the FLP recombinase. For thermal induction of FLP synthesis and subsequent removal of the temperature-sensitive plasmid pCP20, mutants were incubated at 37°C.

To generate YP73 and YP75, a Δ*ymoA* fragment was inserted into YP69 (YPIII Δ*csrB*) and YP72 (YPIII Δ*rovM*), respectively. First, the kanamycin resistance gene was amplified using primer pairs 25/26 for YP73 and YP75 (see Supplementary Table 3). Next, the *Yersinia* genomic DNA was used as a template to amplify 500-bp regions flanking the target genes *ymoA* (see Supplementary Table 3). The upstream fragment was amplified with a primer pair of which the reverse primer contained additional 20 nt at the 5′-end which were homologous to the start of the kanamycin resistance gene, the downstream fragment was amplified with a primer pair of which the forward primer contained additional 20 nt at the 5′-end which were homologous to the end of the kanamycin resistance gene (for primer see Supplementary Table 4). In the next step, a PCR reaction was performed with the forward primer and the reverse primer using the upstream and downstream PCR products of the target gene and the *kan* gene fragment as templates. The PCR fragment was transformed into *Y. pseudotuberculosis* YPIII pKD46 and chromosomal integration of the fragments was selected by plating on LB supplemented with kanamycin. The selection of the mutants and removal of the kanamycin resistance gene was performed as described (Datsenko and Wanner, 2000).

### RNA Isolation and Northern Detection

Overnight cultures were grown to stationary phase (OD_600_ of 3). 2.5 ml culture were withdrawn, mixed with 0.2 volume of stop solution (5% water-saturated phenol, 95% ethanol) and snap-frozen in liquid nitrogen. After thawing on ice, bacteria were pelleted by centrifugation (2 min, 14.000 rpm, 4°C), and RNA was isolated using the SV total RNA purification kit (Promega) as described by the manufacturer. RNA concentration and quality were determined by measurement of *A*_260_ and *A*_280_. Total cellular RNA (10 μg) was mixed with loading buffer (0.03% bromophenol blue, 4 mM EDTA, 0.1 mg/ml EtBr, 2.7% formaldehyde, 31% formamide, 20% glycerol in 4 × MOPS buffer) and was separated on agarose gels (1.2%), transferred overnight onto positively charged membranes (Roche) in 20 × SSC and UV cross-linked. Prehybridization, hybridization to DIG-labeled DNA probes and membrane washing were conducted using the DIG luminescent Detection kit (Roche) according to the manufacturer’s instructions. The *csrC* and *csrB* transcripts were detected with a DIG-labeled PCR fragment (DIG-PCR nucleotide mix, Roche) with primer pair 23/24 and 25/26 (Supplementary Table 2), respectively.

### RNA Stability Assay

RNA stability assay was used to compare degradation of the CsrC RNA between wild-type and different mutant strains. A YPIII overnight culture was mixed with rifampicin at a final concentration of 500 μg/ml to inhibit transcription. At certain time points after blockage of transcription, samples were withdrawn and total RNA was prepared for Northern blot analysis as described above. The half-life of CsrC was calculated by least squares analysis of semi-logarithmic plots of RNA concentration versus time.

### Primer Extension Analysis to Test CsrB and CsrC Stability

Primer extension analysis was performed to determine the steady-state level and the stability of the CsrB and CsrC RNA from strains YPIII and YP80 (YPIII Δ*hfq*). At an OD_600_ of 2.0 (early stationary phase), rifampicin was added to a final concentration of 500 μg ml^–^, after 0, 10, 20, 30, and 60 min, 2 ml aliquots were withdrawn and total RNA was extracted of the samples using the SV total RNA purification kit (Promega) as described by the manufacturer. Annealing was performed with 5 μg extracted RNA and the 5′-Dig-labeled oligonucleotides (primer 5′-CTGAAGACACATCTTCC-3′ for CsrB, primer 5′- CCTGAGTAACTGTGCTCC-3 for CsrC, and primer 5′-CCCACACTACCATCGGCGC-3′ for 5S RNA) in 20 μl of 1x First Strand Buffer (Invitrogen) by slow cooling of the sample (0.01°C/sec) including 8 mM dNTPs with 200 U Superscript II reverse transcriptase (Invitrogen) was added and incubated for 1 h at 42°C. The size of the Dig-labeled reaction products was determined on a denaturing 4% DNA sequencing gel by a detection procedure as described (Heroven et al., 2008).

### Expression and Purification of the *Y. pseudotuberculosis* YmoA and H-NS Protein

KB4 (Δ*hns*, Δ*stpA*, and Δ*hha*) transformed with pAKH77 or pAKH11 was grown at 37°C in LB broth to an *A*_600_ of 0.6. Anhydrotetracycline was added (0.2 μg/ml) to induce the expression of YmoA-Strep-Tag and/or 2 mM IPTG was used to induce H-NS-His_6_ expression. For purification of the YmoA-H-NS heterodimer KB4 transformed with pAKH77 and pAKH11 was used for overexpression of the YmoA and H-NS protein. The cells were grown for an additional 3 h before being harvested. The purification procedure for the Strep-tagged YmoA protein was performed according to the manufacturer’s instructions (IBA GmbH, Germany). H-NS purification was performed as described (Heroven et al., 2004). The purity of the YmoA and the H-NS protein was estimated to be >95%.

### RNA/DNA Retardation Assays

For RNA-binding studies the purified YmoA and H-NS proteins were dialyzed against the RNA-binding buffer (10 mM Tris-HCl pH 7.5; 3 mM M DTT; 7,5% glycerol; 100 mM KCl; 100 mM MgCl_2_). The CsrC RNA for RNA band shift analysis was obtained by *in vitro* transcription from a PCR fragment as described (Heroven et al., 2008; Böhme et al., 2012). The *csrC* transcript (extending 150 nt downstream of the transcriptional start site of *csrC*) and the control transcript (extending 117 nt downstream of the transcriptional start site of the 5S rRNA gene) were synthesized *in vitro* using the Fermentas TranscriptAid T7 High Yield Transcription Kit from PCR products of the target regions. Primers 29/30 and 31/32 were used for amplification of the *csrC* and 5S RNA control fragment from chromosomal DNA of YPIII. The RNA transcripts were extracted with phenol:chloroform, precipitated with ethanol and stored in DEPC-treated water. The RNA-binding reactions included the CsrC (1.1 μM) and 5S rRNA transcripts (1 μM), 1× RNA-binding buffer and increasing concentrations of the H-NS and YmoA proteins. In the following, they were incubated for 30 min at room temperature and immediately loaded on 8% polyacrylamide gels.

For DNA-binding studies, the purified YmoA and H-NS proteins were dialyzed against the DNA-binding buffer (10 mM Tris-HCl, pH 7.5, 1 mM EDTA, 5 mM dithiothreitol, 5% glycerol, 10 mM NaCl, 1 mM MgCl_2_, 100 μg/ml BSA). Defined PCR fragments, carrying portions of the *csrC* regulatory region and the *csiD* region of *E. coli* K-12 CC118λpir (negative control), were mixed in an equimolar ratio and incubated with increasing amounts of purified YmoA and/or H-NS for 20 min at room temperature and used for DNA band shift assays as described by (Böhme et al., 2012).

### Microarray Analysis and Data Analysis

Sequences used for the design of the microarrays (Agilent, 8 × 15K format), containing three different 60 nt oligonucleotides for all 4,172 chromosomal genes (ORFs > 30 codons) of the *Y. pseudotuberculosis* YPIII genome and six probes for the 92 genes of the virulence plasmid pYV of *Y. pseudotuberculosis* strain IP32953, were obtained from the NCBI Genome Genbank (NC_010465 and NC_006153). The ORF-specific oligonucleotides were designed using the web design application eArray from Agilent^1^. 16 independent cultures of *Y. pseudotuberculosis* YPIII and the *ymoA* mutant strain were grown in LB at 25°C to OD_600_ 0.8, total RNA was isolated from samples using the SV total RNA purification kit (Promega), and RNA concentration and quality was determined with an Agilent 2100 Bioanalyzer using the RNA Nano6000 kit as described by the manufacturers. Total RNA of four independent samples was pooled. 1 μg of the pooled samples was used for RNA-labeling with Cy5 (for wild-type RNA) and Cy3 (for mutant RNA) using the ULS^TM^ Fluorescent Labeling Kit for Agilent Arrays (Kreatech). Non-incorporated Cy5/Cy3 was removed by KREA*pure* purification columns as suggested by the manufacturers. The degree of labeling was determined by a Nanodrop (Peqlab). Subsequently, 300 ng Cy5-labeled RNA and 300 ng Cy3-labeled RNA were mixed, fragmented and hybridized to custom-made Agilent microarray slides (8 × 15K) using the Agilent gene expression hybridization kit as described by the manufacturer. Direct use of labeled RNA for array hybridization was chosen to avoid bias obtained by cDNA library formation. In general, four biological replicates were used for each experiment. After washing and drying of the microarray slide, data were scanned using Axon GenePix Personal 4100A scanner and array images were captured using the software package GenePix Pro 6.015.

The processing of the resulting microarray data was performed using the software package R^2^ in combination with the ‘‘Bioconductor’’ software framework^3^ (Gentleman et al., 2004). Preprocessing based on the marray package employing the read.GenePix function. Control code for probe selection was adapted to the custom-made Agilent microarray system and a quality control was performed to check for hybridization artifacts and large-scale differences between the microarrays of one experiment. A two-color intensity-dependent normalization (“Lowess” normalization) was applied and if necessary supplemented by scale normalization between different microarrays as described (Yang et al., 2002). Differentially expressed genes were obtained using the limma package and the lmFit function for linear modeling. eBayes was used for significance calculations (Smyth, 2004). The overall fold-changes of a gene represented by at least three probes are given as median values for all probes. A cut-off of fold-change ≥ 2 (*P*-value of >0.001) was chosen to determine YmoA-dependent genes/operons and the set of resulting differential expressed genes was analyzed employing the topGO package for Gene Ontology (GO) term enrichment (Alexa et al., 2006). MIAME compliant array data were deposited in Gene Expression Omnibus (GEO) database and are available via the following accession number: GSE35043.

### Quantitative RT-PCR

One step real-time RT-PCR was performed in triplicate with RNA preparations of six independent cultures using a Rotor-Gene Q thermo cycler (Qiagen). For qRT-PCR analysis RNA was prepared from bacterial cells grown to early stationary phase at 25°C using Qiazol and the chloroform/phenol extraction method. Quantitative RT-PCR was carried out with the SensiFast SYBR No-ROX One-Step Kit (Bioline, Germany) applying the 3-step cycling protocol according to the manufacturer. Gene specific-primers used for qRT-PCR amplification are listed in Supplementary Table 3 and were designed to produce a 200–250 bp amplicon with *Y. pseudotuberculosis* YPIII cDNA as template. The amount of PCR product was quantified by measuring fluorescence of SYBR Green dye. Reported gene expression levels were normalized to levels of the *sopB* transcript. This gene was used as it exhibited identical expression levels in the wild-type and the *ymoA* mutant under used experimental conditions. Standard curves were detected during every run for each gene tested and established by comparing transcript levels in serial dilutions of total RNA from a control sample. The relative expression of each gene was calculated as described (Pfaffl, 2001).

### β-Galactosidase and Alkaline Phosphatase Assays

The activity of the *inv-phoA* fusion encoded on plasmid pPD297 and the β-galactosidase activity of the *lacZ* fusion constructs were measured in permeabilized cells as described previously (Manoil, 1990; Miller, 1992). The activities were calculated as follows: β-galactosidase activity OD_420_ ⋅ 6.75 ⋅ OD_600_^–1^ ⋅ Δ*t* (min)^–1^ ⋅ Vol (ml)^–1^; alkaline phosphatase activity OD_420_ ⋅ 6.46 ⋅ OD_578_^–1^ ⋅ Δ*t* (min)^–1^ ⋅ Vol (ml)^–1^.

### Gel Electrophoresis, Preparation of Cell Extracts and Western Blotting

For immunological detection of the YmoA, RovA, RovM, and invasin proteins, *Y. pseudotuberculosis* cultures were grown under specific environmental conditions as described. Cell extracts of equal amounts of the bacteria were prepared and separated on a 15% (RovA, Hfq), 12% (RovM) or 10% (invasin) SDS-PAGE (Sambrook, 2001). The low molecular weight proteins (YmoA and CsrA) were separated by 20% TRICINE-PAGE as described recently (Schagger, 2006).

Subsequently, the samples were transferred onto an Immobilon-P membrane (Millipore) and probed with a polyclonal antibodies directed against RovA, RovM, Hfq, CsrA, and YmoA, or a monoclonal antibody 3A2 directed against invasin as described recently (Heroven and Dersch, 2006). The cell extracts used for Western blotting were also separated by SDS-PAGE and stained with Coomassie blue to ensure that the protein concentrations in the different cell extracts are comparable; about 10 μg protein was applied of each sample.

### Cell Invasion Assay

In preparation of the cell adhesion and uptake assay, 5 x 10^4^ HEp-2 cells were seeded and grown overnight in individual wells of 24-well cell culture plates (Nunc). Cell monolayers were washed three times with phosphate-buffered saline (PBS) and incubated in binding buffer (RPMI 1640 medium supplemented with 20 mM HEPES pH7.0 and 0.4% bovine serum albumin) before infection with bacteria. Approximately 10^6^ bacteria were added to the monolayer and incubated at 37°C. Bacterial uptake was assessed 30 min after infection as the percentage of bacteria, which survived a gentamicin treatment versus the input cell number as described previously (Dersch and Isberg, 1999). The experiments were routinely performed in triplicate.

### Mouse Infections

*Yersinia pseudotuberculosis* YPIII (wild-type) and YP50 (*ymoA* mutant) were grown overnight at 25°C, washed in sterile PBS and used for intragastrical inoculation of 6–8 weeks old female BALB/c mice (Janvier, France) using a ball-tipped feeding needle. To assess the impact of an *ymoA* deletion in *Y. pseudotuberculosis* on tissue colonization, different groups of BALB/c mice were infected with 5 × 10^8^ bacteria of each strain. In co-infection experiments mice were orally inoculated with an equal mixture of strains YPIII and YP50, each at a dosage of 5 × 10^8^ CFU. Three days post infection, mice were sacrificed using CO_2_. Mesenterial lymph nodes, liver and spleen were recovered. Isolated Peyer’s patches were washed with sterile PBS, incubated in 100 μg/ml gentamycin for 30 min and washed intensively with sterile PBS three-times. Organs were weighed and homogenized for 30 s in sterile PBS using a Polytron PT 2100 homogenizer (Kinematica, Switzerland). Subsequently, they were plated on *Yersinia* selective agar (Oxoid, Germany) in three serial dilutions with or without kanamycin. Colony forming units were determined and are given in cfu per gram organ/tissue. The competitive index in comparison to the wild-type strain YPIII was calculated as described (Monk et al., 2008).

### Ethics Statements

Animal work was performed in strict accordance with the German regulations of the Society for Laboratory Animal Science (GV-SOLAS) and the European Health Law of the Federation of Laboratory Animal Science Associations (FELASA). The animal study was reviewed and approved by the Niedersächsisches Landesamt für Verbraucherschutz und Lebensmittelsicherheit: animal licensing committee permission no. 33.9.42502-04-055/09.

## Results

### The Modulator YmoA Has a Global Influence on Gene Expression of *Y. pseudotuberculosis*

In a previous study we showed that YmoA directly represses transcription of the regulator of *lcrF*, which is encoded on the *Yersinia* virulence plasmid (pYV) and controls expression of the antiphagocytic T3SS machinery and effectors (Böhme et al., 2012). However, despite its influence on *lcrF* expression, the overall influence of YmoA on *Y. pseudotuberculosis* virulence was still unknown. In the context of a previous study addressing the influence of different early-stage virulence regulators (CsrA, Crp, and RovA) on gene expression of *Y. pseudotuberculosis* YPIII (Bücker et al., 2014), we also investigated the effect of YmoA on the *Yersinia* transcriptome. The analysis was performed with *Y. pseudotuberculosis* wildtype strain YPIII and the isogenic *ymoA* mutant (YP50) grown at 25°C to late exponential phase, conditions under which YmoA production was found to be high (Supplementary Figure 1). In total 289 genes showed twofold or greater difference in transcript abundance between the wild-type and the Δ*ymoA* strain. Among them, 227 genes (79%) were up-regulated and 62 (21%) were down-regulated in the *ymoA* mutant (Supplementary Table 4), indicating that YmoA is an important global regulator implicated in the control of fitness- and virulence-relevant processes. Classification according to genome annotation of *Y. pseudotuberculosis* YPIII showed that altered genes belong to several functional categories (Figure 1A and Supplementary Table 4). About 31% of all YmoA-dependent transcripts (90 genes) are related to virulence. As expected from previous studies (de la Cruz et al., 1992; Cornelis, 1993), 42 genes of the virulence plasmid encoding the structural components of the type III secretion system (*yscA-L*, *yscO-S*, and *yscU*), the intracellularly delivered Yop effector proteins and their chaperones (*yopE, sycE, yopH, yopJ, yopO, sycO, yopK*, and *yopM*), regulators of the secretion system (*yscW-lcrF, lcrQ, yopN-sycN-tyeA-yscXY*, and *lcrDRGVH-yopBD*), and the adhesin YadA were upregulated in the *ymoA* mutant. Notably, multiple virulence plasmid-encoded putative resolvases and transposase genes were also upregulated in the *ymoA* mutant (Supplementary Table 4), suggesting that YmoA did not only control expression of the T3SS-Yop injectisome via its influence on LcrF (Böhme et al., 2012), but seems to have a more global effect.

In addition, many chromosomally encoded virulence genes were differentially regulated in the absence of YmoA. Among them were genes for potential adhesins (*ail*, *ailB*, *psaA, yadC*, and *yadF*) and fimbriae/pili (*ybgP, fimA1-3, fimC, fimD, smfA1-2*, and *csgG*), the *cnfY* gene encoding a cytotoxic necrotizing factor and the insecticidal toxin genes *tcaAB*, as well as genes for iron/heme acquisition (*hasA* and *bfr*), LPS/O-antigen synthesis (*wzz, manB, gne, wbyL, manC, fcl, gmd*, and *wbyK*) and autoinducer synthesis (*lsrRBFG, ypsI/ytbI*). In addition, genes encoding Hcp1 family effectors and different operons for type VI secretion systems (*hcp1* and *imp* genes), flagella assembly and chemotaxis genes (*flhDC, fliETFZ*, and *flgDCB*) were upregulated in the *ymoA* deficient strain (Figures 1A,B and Supplementary Table 4). These data were validated with a subset of identified YmoA-dependent genes using qRT-PCR with *sopB* as control as *sopB* was not affected by YmoA according to the microarray analysis [Figure 1C, *YPK_0604* (*l-psp*; 9.3-fold), *grpE* (3-fold), *dnaJ* (4.8-fold), and *dnaK* (2.8-fold)].

### YmoA Controls the CsrABC-RovM-RovA Regulator Signaling Cascade in *Y. pseudotuberculosis*

**FIGURE 2:**
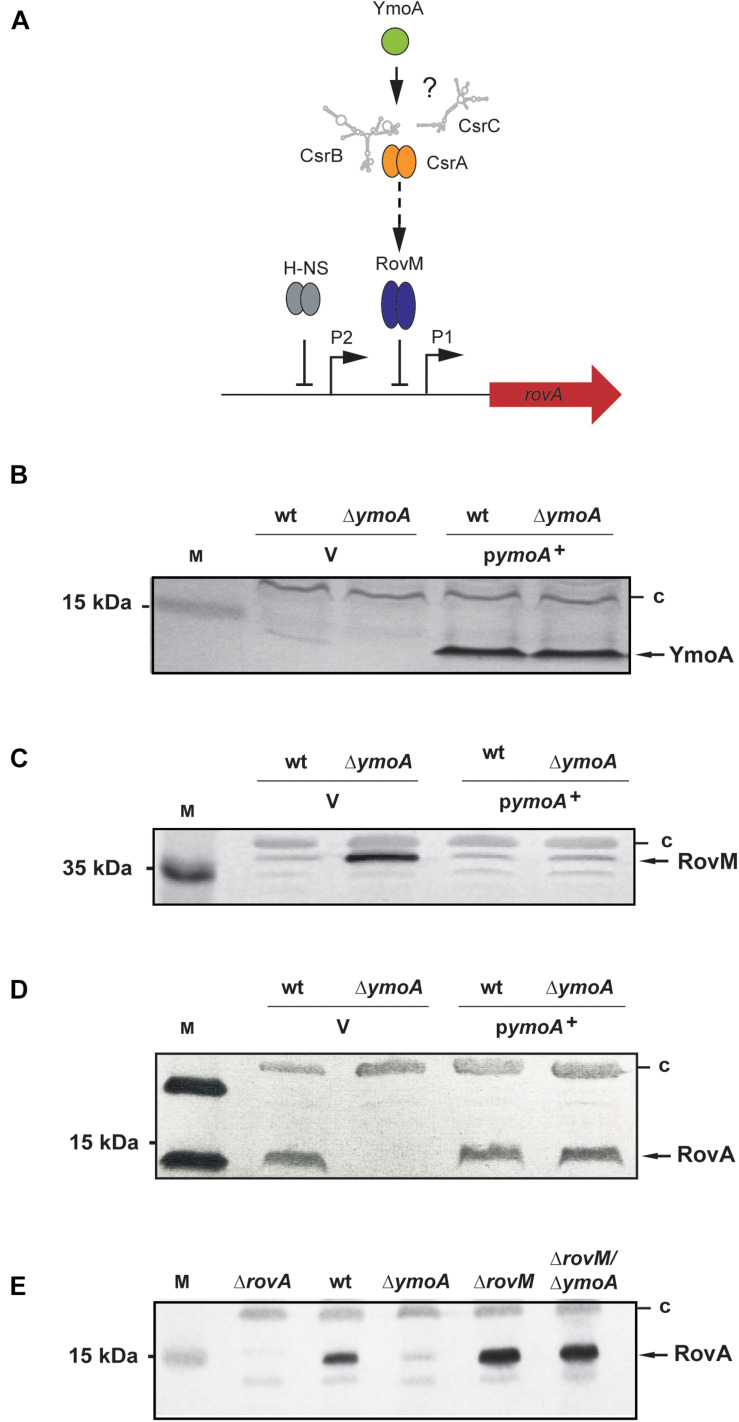
Analysis of RovA and RovM levels in *Y. pseudotuberculosis* in the presence or absence of YmoA. **(A)** Regulatory components controlling *rovA* expression in *Y. pseudotuberculosis* are illustrated and assumed influence of YmoA on the regulatory cascade is indicated by ‘?’. **(B–D)** Whole-cell extracts from overnight cultures of *Y. pseudotuberculosis* wild-type and mutant strain YP50 (Δ*ymoA*) transformed with pACYC184 (V) or pAKH71 (*ymoA*^+^) grown at 25°C were prepared and analyzed by Western blotting with a polyclonal antibody directed against YmoA **(B)**, RovM **(C)**, and RovA **(D)**. A molecular weight marker is loaded on the left. **(E)** Whole-cell extracts from overnight cultures of the *Y. pseudotuberculosis* wild-type (YPIII), and the *rovA* (YP3), *ymoA* (YP50), *rovM* (YP41), and *rovM/ymoA* (YP73) mutant strains were prepared. RovA in the extracts were detected by Western blotting using polyclonal antibodies directed against these proteins. A prestained molecular weight marker is loaded on the left. Unspecifically detected proteins (c) were used as loading control.

Among the identified YmoA-dependent virulence genes were early-stage pathogenicity factors of *Yersinia*, which are predominantly expressed at lower temperature *in vitro*. Several of them, including the virulence regulator gene *rovA* (Supplementary Table 4), have been shown to be controlled by the Csr system (Heroven et al., 2008, 2012a; Bücker et al., 2014). This suggested that YmoA might be implicated in the control of the CsrABC-RovM-RovA regulatory cascade (Figure 2A). To prove this assumption, we first compared intracellular YmoA, RovM and RovA levels between wild-type (YPIII), the *ymoA* mutant (YP50) and the complemented strain YP50 (Δ*ymo*A) pAKH71 (*ymo*A^+^). As shown in Figures 2B–D, expression of the negative regulator RovM is significantly increased in the absence of YmoA, which in turn leads to a strong reduction of the amount of RovA. Influence on RovM and RovA levels could be fully complemented by the *ymoA*^+^ plasmid. Interestingly, the intracellular amount of YmoA is very low in *Y. pseudotuberculosis* YPIII under growth conditions simulating the early-stage of infection (Figure 2B and Supplementary Figure 1), but this concentration seems sufficient to promote RovA synthesis to levels similar to p*ymoA*^+^ containing strains (Figure 2D), with significantly higher amounts of YmoA (Figure 2B).

While a deletion of *ymoA* alone abrogated RovA synthesis, it had no influence on *rovA* expression in the *rovM/ymoA* double mutant; in the contrary higher RovA level were detected similar to the *rovM* mutant (Figure 2E). This demonstrated that YmoA exerts is effect mainly via RovM. This was somewhat surprising as YmoA was found to interact with the nucleoid-structuring protein H-NS (Nieto et al., 2002) which is known to control *rovA* expression directly (Heroven et al., 2004) (Figure 2A). In fact, overexpression of a dominant-negative N-terminal H-NS fragment (H-NS^∗^) – a knock-out is deleterious to *Y. pseudotuberculosis –* reduced H-NS-mediated *rovA* silencing as shown previously (Heroven et al., 2004). This led to an increase of RovA levels in the *Y. pseudotuberculosis* wild-type, but not in the *ymoA* mutant (Supplementary Figure 2).

**FIGURE 3:**
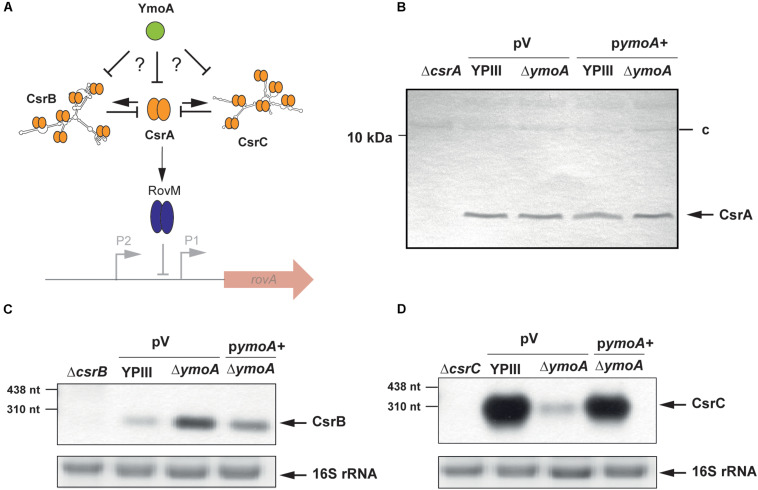
Influence of YmoA on the synthesis of CsrA and the Csr-type RNAs of *Y. pseudotuberculosis.* **(A)** Illustration of potential YmoA influence on RovM synthesis via the CsrABC system. **(B)** Whole-cell extracts from overnight cultures of *Y. pseudotuberculosis* wild-type and mutant strain YP50 (Δ*ymoA*) transformed with pACYC184 (V) or pAKH71 (*ymoA*^+^) grown at 25°C were prepared and analyzed by Western blotting with as a polyclonal antibody directed against CsrA YP50 (Δ*ymoA*). YP53 (Δ*csrA*) was used a negative control, and an unspecifically detected protein (c) served as loading control. **(C,D)** Total RNA of *Y. pseudotuberculosis* YPIII and YP50 (Δ*ymoA*) carrying the empty vector pAKH85 (V) and YP50 pAKH71 (p*ymoA*^+^), YP51 (Δ*csrB*) and YP48 (Δ*csrC*) was prepared, separated on agarose gels and a CsrB- or CsrC-specific probe was used to detect CsrB **(C)** or CsrC **(D)** by Northern blotting. The 16S rRNAs are shown as RNA loading control. Used RNA marker is indicated on the left.

Expression of the *rovM* gene was shown to be under control of the *Yersinia* Csr system, comprising the two regulatory ncRNAs, CsrB, and CsrC, and the RNA-binding protein CsrA (Heroven et al., 2008) (Figure 3A). The CsrB and CsrC RNAs both sequester the CsrA RNA-binding protein resulting in reduced RovM synthesis. To determine whether YmoA acts on *rovM* via the *Yersinia* Csr system, we compared the amount of CsrA and expression of the *csr* RNA genes between wild-type and the *ymoA* mutant. While no changes of CsrA levels were detectable (Figure 3B), a mild increase in CsrB and a strong reduction of CsrC could be observed in a Δ*ymoA* strain compared to the wild-type (Figures 3C,D).

### YmoA Has a Positive Effect on CsrC Levels

As both Csr-RNAs have a negative influence on each other (Heroven et al., 2008), we first addressed whether YmoA affects only the expression of CsrC RNA, which in turn inhibits the synthesis of CsrB. In fact, identical changes were observed on CsrC and RovM levels in a *ymoA* and a *csrB/ymoA* mutant strain, implying that YmoA exerts its major effect on the Csr regulon through regulation of CsrC (Supplementary Figure 3).

**FIGURE 4:**
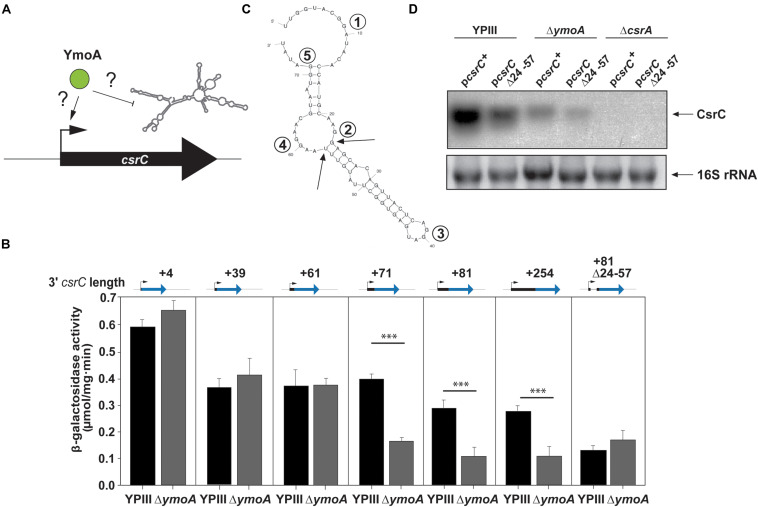
YmoA-mediated control of CsrC occurs on the post-transcriptional level. **(A)** Model for YmoA influence on CsrC on the transcriptional or post-transcriptional level. Increased CsrC levels can result from increased transcription or decreased degradation of CsrC. **(B)** Plasmids harboring transcriptional *csrC-lacZ* fusions with varying 3′-ends of the *csrC* gene and a *csrC*(+81)-*lacZ* fusion with a Δ(+24–+57) deletion (pKB17) were transformed into *Y. pseudotuberculosis* YPIII and YP50 (Δ*ymoA*). Strains were grown overnight at 25°C and transcriptional activity of the different fusions was determined by β-galactosidase assays. β-galactosidase activity is given in μmol min^–1^ mg^–1^ for comparison. The data represent the average ± SD from at least three different experiments each done in duplicate. The statistical significances between the wild-type and the *ymoA* mutant were determined by the ANOVA test. *P*-values: ^∗∗∗^: <0.001. **(C)** Secondary structure prediction of the CsrC RNA from nucleotide +1 to +76 generated by Mfold. The single-stranded regions including the most highly conserved GGA element of CsrA binding sites are indicated by numbers. The arrows indicate the 5′ and 3′ end of the Δ(+24–+57) deletion. **(D)** *Y. pseudotuberculosis* strains YPIII (wild-type), YP50 (Δ*ymoA*), and YP53 (Δ*csrA*) harboring pKB59 (*csrC*^+^) or pKB49 (*csrC*Δ+24–+57) were grown in LB at 25°C overnight. Total RNA of the strains was extracted, separated on agarose gels and a CsrC-specific probe was used to detect the CsrC RNA by Northern blotting. The 16S rRNAs are shown as RNA loading control.

To determine whether this positive effect of YmoA on CsrC occurs on the transcriptional or post-transcriptional level (Figure 4A), we analyzed expression of *csrC-lacZ* transcriptional fusions harboring *csrC* fragments with varying 3′ endpoints (+4, +39, +61, +71, +81, or +254). In general, elongation of the *csrC* fragment led to a continuous decrease of *csrC-lacZ* expression (Figure 4B), suggesting that *csrC* sequences destabilize or reduce translation of the *lacZ* RNA and/or lead to a premature termination of transcription. Furthermore, we found that absence of YmoA had no significantly different effect on *csrC-lacZ* reporter fusions which included the first ≤61 nucleotides of *csrC*, whereas expression of fusions, containing the first 71 nucleotides or more, was more than twofold reduced. Computational secondary structure analyses of the *csrC* transcript predict that nucleotides from position +24 to +57 form a hairpin structure (Figure 4C). Expression of mutant variants of *csrC*(+81)-*lacZ* fusion and *csrC* in which nucleotides +24 to +57 were deleted became independent of YmoA (Figures 4B,D). This suggests the presence of (i) a stabilizing element or (ii) a transcriptional terminator within the 5′-sequences of the CsrC RNA which is affected by YmoA. The fact that (i) the 5′-hairpin region has a positive influence on the overall expression of *csrC* and (ii) truncated CsrC species could not be detected in the *ymoA* mutant suggested that YmoA enhances CsrC RNA stability through the stem loop segment. To confirm YmoA-mediated post-transcriptional control of CsrC, *csrC* was expressed under the control of a plasmid-encoded tetracycline promoter (P*_*tet*_*). To ensure expression was only derived from the plasmid, culturing conditions were chosen under which the chromosomal *csrC* gene is fully repressed (Heroven et al., 2008). While expression of the control gene *lacZ* under P*_*tet*_* control was identical in the wildtype and the *ymoA* mutant (Supplementary Figure 4A), considerably higher levels of CsrC were detectable in the wild-type when expressed from the identical P*_*tet*_* expression plasmid (Supplementary Figure 4B). This also suggested that YmoA acts on CsrC on the post-transcriptional level.

**FIGURE 5:**
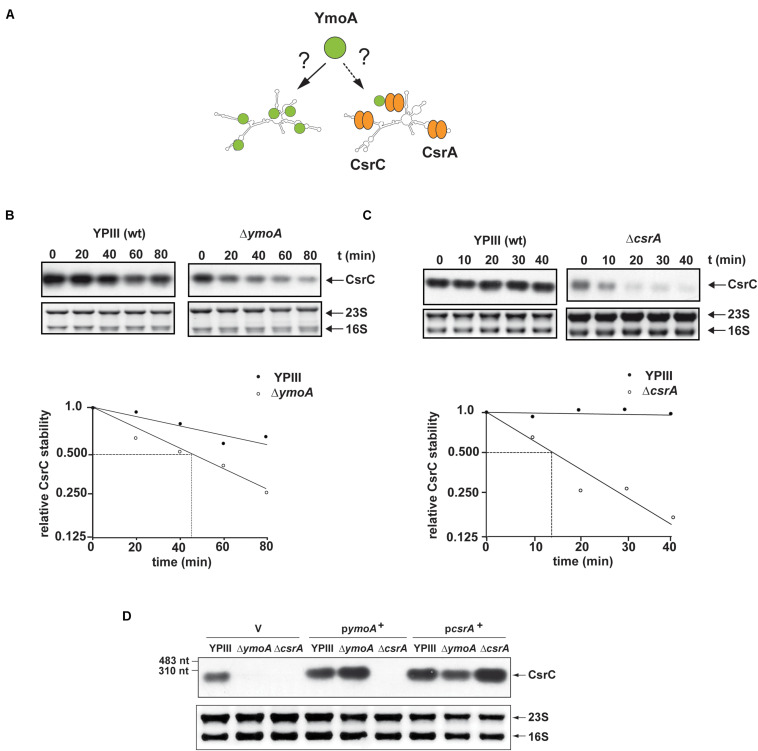
YmoA and CsrA increase the stability of the CsrC RNA. **(A)** Model for YmoA-dependent CsrC stabilization. **(B,C)** The stability of the CsrC RNA expressed under the control of the *tet* promoter was compared between *Y. pseudotuberculosis* strain YPIII (wild-type) and YP50 (Δ*ymoA*) **(B)** or YP53 (Δ*csrA*) **(C)** and CsrC RNA stability was analyzed over time by Northern blotting with a CsrC- specific probe. A representative Northern blot of three independent experiments is shown in the upper panel. 16S and 23S rRNA were used as loading controls. The band corresponding to the CsrC transcript was quantified using the BioRAD Image Lab^TM^ Software and was plotted versus time of extraction (in minutes) to calculate the half-life of the sRNA indicated by dotted lines (lower panel). **(D)** *Y. pseudotuberculosis* strains YPIII, YP50 (Δ*ymoA*), and YP53 (Δ*csrA*) harboring vector pHSG576 (V), a *ymoA*^+^ (pKB4) or *csrA*^+^ derivative (pKB60) were cultivated overnight in LB medium at 25°C. Total RNA of the strains was extracted, separated on agarose gels and a CsrC-specific probe was used to detect the CsrC ncRNA by Northern blotting. Used RNA marker is marked on the left. The 16S and 23S rRNAs are shown as RNA loading control.

To further analyze the influence of YmoA on CsrC (Figure 5A), CsrC levels were determined by Northern blotting after transcription of P*_*tet*_*:*csrC* was blocked by rifampicin. As shown in Figure 5B, the CsrC RNA was slowly degraded in the *Y. pseudotuberculosis* wild-type strain with a half-life of about 100 min, whereas CsrC was less stable in the *ymoA* mutant and decayed with a half-life of about 45–50 min. Presence of five potential CsrA-binding sites (GGA motif) within the 5′-hairpin region of the CsrC transcript (Figure 4C), suggested that the interaction and sequestration of CsrA could also increase the stability of the CsrC RNA. Northern blot analysis further revealed that CsrA is important for CsrC abundance as CsrC is not detectable in the Δ*csrA* mutant (Figure 4D). To further characterize CsrA influence on CsrC stability, we induced *csrC* expression under the control of P*_*tet*_* by anhydrotetracycline (AHT). After transcription was blocked by rifampicin, the CsrC transcript was much more rapidly degraded in the *csrA* mutant compared to wildtype (Figure 5C). In comparison, the influence of CsrA on CsrC stability was much more pronounced than that of YmoA (Figures 5B,C). Hence, it seemed possible that presence of YmoA influences the function of CsrA. To test this assumption, we tested whether CsrA overexpression is able to complement a *ymoA* mutant strain or whether YmoA overproduction can complement *csrA* deficiency by comparing CsrC levels in the different recombinant strains. As shown in Figure 5D, overexpression of YmoA was not able to complement *csrA* deficiency, whereas CsrC RNA synthesis was partially complemented when CsrA was overproduced in the *ymoA* mutant. This suggested that loss of YmoA can be overcome by an increase of CsrA-binding to the CsrC RNA. Based on these results, it is possible that YmoA has an influence on the activity of CsrA, e.g., by controlling an RNA chaperone assisting in CsrA-binding or folding of the CsrC RNA.

### The RNA Chaperone Hfq Acts Differently and Independently of YmoA on CsrC

**FIGURE 6:**
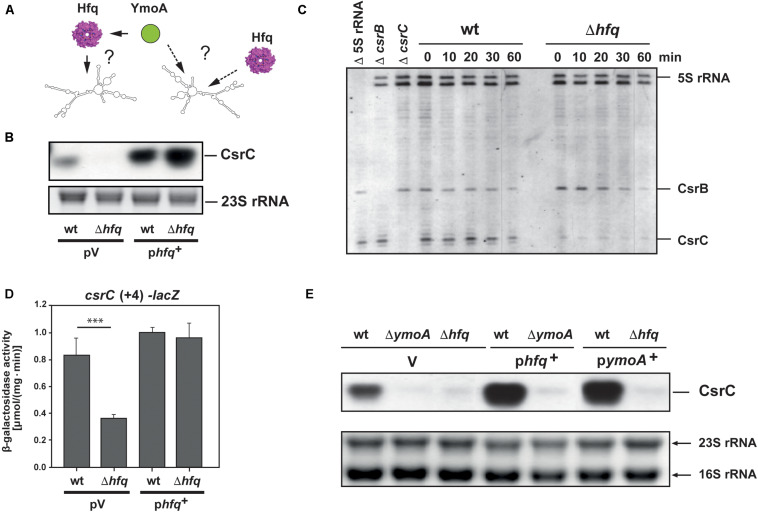
Hfq influence on CsrB and CsrC stability and cooperation with YmoA. **(A)** The model illustrates possible influence of YmoA on CsrC abundance via or independent of Hfq. **(B)** *Y. pseudotuberculosis* strain YPIII (wt) and YP80 (Δ*hfq*) carrying the vector pAKH85 (pV) or its *hfq*^+^ derivative (pAKH115) were grown overnight in LB medium. Total RNA of the samples was isolated, separated on agarose gels and the CsrC RNA was detected by Northern blotting using a CsrC-specific probe. The 23S rRNAs is shown as RNA loading control. **(C)** *Y. pseudotuberculosis* strain YPIII (wt) and YP80 (Δ*hfq*) were grown in LB at 25°C to early stationary phase, rifampicin was added in a final concentration of 500 μg ml^–1^ and aliquots were withdrawn at indicated time points. Total RNA was extracted and used for primer extension analysis. Samples without addition of labeled primers for 5S rRNA (Δ 5S rRNA), *csrB* (Δ*csrB*), and CsrC (Δ*csrC*) were used as controls. The reaction products were separated on 4% DNA sequencing gels. The positions of the transcripts are indicated. **(D)** *Y. pseudotuberculosis* strain YPIII (wt) and YP80 (Δ*hfq*) carrying the vector pAKH85 (pV) or its *hfq*^+^ derivative (pAKH115) and the *csrC-lacZ* fusion (pAKH103) were grown in LB at 25°C overnight. β-Galactosidase activity of the cultures was determined **(upper panels)** and is given in μmol min^–1^ mg^–1^ for comparison. The data represent the average ± SD from at least three different experiments each done in duplicate. The statistical significances between the wild-type and the *ymoA* mutant were determined by the ANOVA test. *P*-values: ^∗∗∗^: <0.001. **(E)** *Y. pseudotuberculosis* strains YPIII, YP50 (Δ*ymoA*) and YP80 (Δ*hfq*) harboring vector pHSG575 (V), a *ymoA*^+^ (pKB4) or *hfq*^+^ derivative (pAKH119) were cultivated overnight in LB medium at 25°C. Total RNA of the strains was prepared, separated on agarose gels and a specific probe was used to detect the CsrC RNAs by Northern blotting. The 16S and 23S rRNAs are shown as RNA loading control.

One molecule that has been shown to assist in folding of Csr/Rsm-type RNAs, CsrA/RsmA binding and increases the stability and abundance of Csr/Rsm-type RNAs in *Pseudomonas aeruginosa* is the ubiquitous hexameric RNA-chaperone Hfq (Sonnleitner et al., 2006; Sorger-Domenigg et al., 2007). Hence, it seemed possible that YmoA exerts its stabilizing influence on CsrC via upregulation of Hfq (Figure 6A). As shown in Figure 6B, no or only very low amounts of the CsrC RNA were detected in an Δ*hfq* mutant strain. This resulted in a dramatic increase of RovM and decrease of RovA levels and all these effects could be complemented by an *hfq*^+^ plasmid (Supplementary Figure 5). To elucidate how Hfq affects the Csr-RNAs, we performed a primer extension analysis to test whether absence of Hfq affects the abundance of CsrB and CsrC when transcription was blocked. Although slightly more CsrB transcript was found in the *hfq* mutant, the CsrB RNA seemed to be more rapidly degraded in the absence of Hfq (Figure 6C). In contrast, overall CsrC levels were significantly reduced in the Δ*hfq* strain, but the half-life of the transcript was not influenced by Hfq (Figure 6C). This strongly indicated that Hfq has a positive influence on *csrC* transcription, but not on CsrC RNA stability. In agreement with this assumption we found that expression of the *csrC*(+4)*-lacZ* fusion harboring only the first four nucleotides of the *csrC* gene was downregulated in the *hfq* mutant (Figure 6D). Complementation assays further demonstrated that neither YmoA nor Hfq could compensate the effect of the *hfq* or the *ymoA* knock-out mutation on the abundance of the CsrC RNA (Figure 6E). Based on this observation, we concluded that Hfq and YmoA act differently and independently on CsrC.

### H-NS Influences CsrC Stability

**FIGURE 7:**
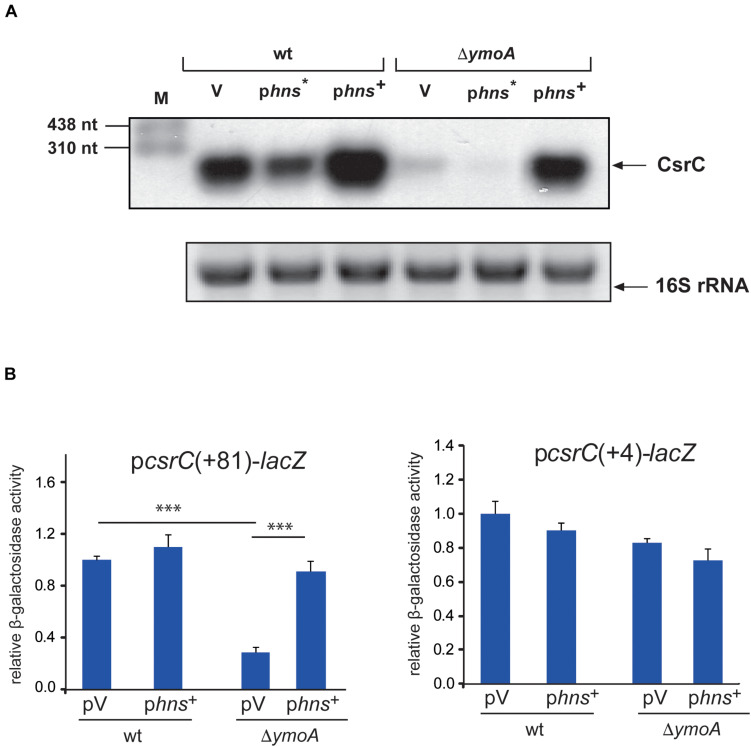
H-NS has a positive influence on *csrC*. **(A)** *Y. pseudotuberculosis* strain YPIII and YP50 (Δ*ymoA*) harboring the empty vector (V), a hns* (pAKH31) or *hns*^+^ (pAKH74) overexpression construct were cultivated overnight in LB medium at 25°C. Total RNA of the strains was prepared, separated on agarose gels and a specific probe was used to detect the CsrC RNA by Northern blotting. The 16S rRNA is shown as RNA loading control. **(B)** Expression of *csrC*(+4)-*lacZ* and *csrC*(+81)-*lacZ* was analyzed in overnight cultures of *Y. pseudotuberculosis* strains YPIII (wild-type) and YP50 (Δ*ymoA*) harboring vector pACYC184, pAKH85 or its *hns*^+^ derivative pAKH74 grown in LB at 25°C. β-galactosidase activity from the overnight cultures was determined and is given as relative expression for comparison. The data represent the average ± SD from at least three different experiments each done in duplicate. Data were analyzed by the ANOVA test, ^∗∗∗^*P* < 0.001.

As members of the YmoA/Hha protein family are able to form heteromeric complexes with H-NS (Nieto et al., 2002), we wanted to know whether YmoA acts through H-NS on CsrC. If so, changes in the active amounts of H-NS should influence CsrC RNA levels in *Y. pseudotuberculosis.* As shown in Figure 7A, the intracellular CsrC level was reduced in the presence of the dominant negative *hns*^∗^ allele and increased upon *hns* overexpression. Moreover, low abundance of CsrC in the *ymoA* mutant was further reduced by *hns*^∗^, whereas overexpression of H-NS is able to compensate for the loss of YmoA and led to CsrC levels similar to the wild-type strain (Figure 7A). Furthermore, a significant reduction of *csrC*(+81)-*lacZ* expression, was observed in the absence of *ymoA* which could be compensated by overexpression of *hns* (Figure 7B). However, the identical changes of YmoA and H-NS levels had no influence on *csrC*(+4)-*lacZ* expression, indicating that both regulatory factors act through the *csrC* fragment including positions +5 to +81.

As loss of YmoA did not influence H-NS levels (Supplementary Figure 6), we first assumed that YmoA enhances H-NS activity through YmoA-H-NS complex formation, and this could have a positive influence on CsrC level. Although YmoA and H-NS were shown to act predominantly on the transcriptional level, it has also been demonstrated that H-NS and paralogous proteins such as StpA can bind specifically to RNA and affect their folding and/or susceptibility to nuclease cleavage by RNases (Zhang et al., 1996; Mayer et al., 2002; Brescia et al., 2004). We tested whether YmoA and/or H-NS of *Y. pseudotuberculosis* could bind to the CsrC RNA *in vitro*. For this purpose, purified CsrC RNA transcript was incubated with increasing concentrations of the *Yersinia* H-NS and/or YmoA proteins. Both proteins were overexpressed and purified from *E. coli* strain KB4 deficient of all *E. coli* H-NS/Hha family proteins. However, neither H-NS alone nor in complex with YmoA was found to bind specifically to the CsrC RNA (Supplementary Figure 7A), whereas specific H-NS-dependent high molecular weight complexes were identified with a *yscW* promoter control fragment as reported previously (Böhme et al., 2012), demonstrating that the H-NS protein is active (Supplementary Figure 7B). This strongly suggests that YmoA and H-NS influence on CsrC is indirect, e.g., occurs through a factor controlling CsrC stability.

### Regulation of CsrC by YmoA and CsrA Depends on Environmental Parameters

**FIGURE 8:**
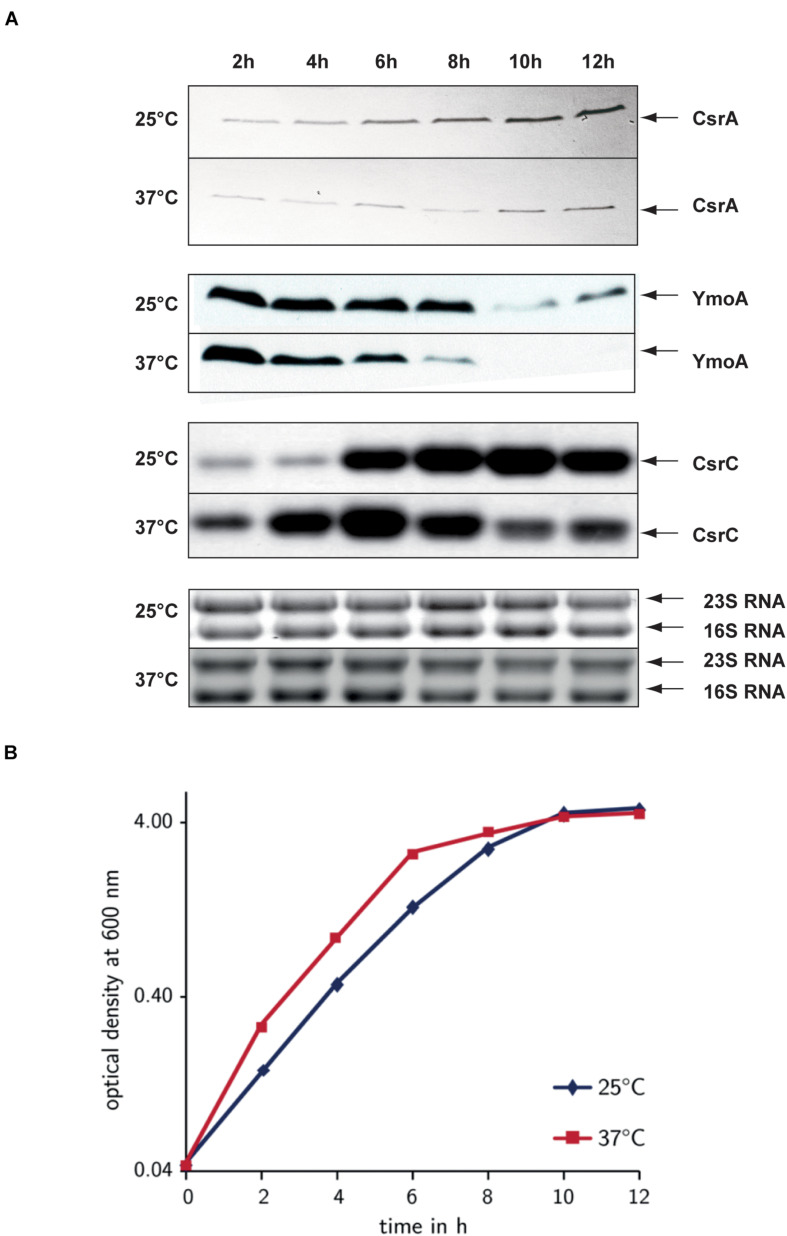
Comparison of the environmental control of CsrA, YmoA, and CsrC synthesis. *Y. pseudotuberculosis* strain YPIII was diluted 1:100 from an overnight culture and was grown in LB at 25 and 37°C. Every 2 h, samples were withdrawn for whole cell extract and total RNA preparations **(A)** and the OD_600_ of the cultures was determined to monitor growth **(B)**. **(A)** Total RNA of the samples was separated on agarose gels and the CsrC RNA was detected by Northern blotting using a CsrC-specific probe. Whole cell extracts were separated on 18% TRICINE polyacrylamide gels and analyzed by Western blotting with a polyclonal antibody directed against YmoA and CsrA. The CsrC RNA band and the YmoA and CsrA protein bands are indicated by arrows. **(B)** OD_600_ of the cultures used for the upper analysis was plotted over time to illustrate and compare the different growth phases.

Next, we analyzed how YmoA and CsrA cooperate in the control of CsrC in response to different environmental parameters. To do so, we monitored the amount of CsrA and YmoA in the wild-type under different growth conditions. Synthesis of CsrA was slightly dependent on growth phase control resulting in moderately higher protein levels during stationary phase, but temperature and media composition had no considerable effect (Supplementary Figure 1). In contrast, YmoA protein levels were strongly dependent on temperature and growth phase, and at 37°C also on nutrient composition of the medium. Maximal amount of YmoA were observed at moderate temperature, in complex medium during exponential growth whereas no YmoA was detectable at 37°C in minimal medium (Supplementary Figure 1). To analyze the influence of the two regulatory factors in more detail, we determined CsrA, YmoA, and CsrC levels along the bacterial growth curve at 25 and 37°C in complex medium. At 25°C, highest CsrC levels were detected during stationary phase (8–12 h), whereas at 37°C CsrC levels were maximal during late exponential phase (6 h), but decreased upon entry into stationary phase (Figure 8). As, the intracellular concentration of CsrA remained at the same or even higher level, the decrease of CsrC amounts seems to result mainly from the strong reduction of YmoA levels during stationary phase, which is more pronounced at 37°C than at 25°C. The latter is the result of temperature-regulated proteolysis of YmoA mainly by the Lon protease but to a minor extent also by the ClpP protease (Supplementary Figure 8), similar to what has been described for the YmoA protein of *Yersinia pestis* (Jackson et al., 2004).

### The Modulator YmoA Is Crucial for Virulence

**FIGURE 9:**
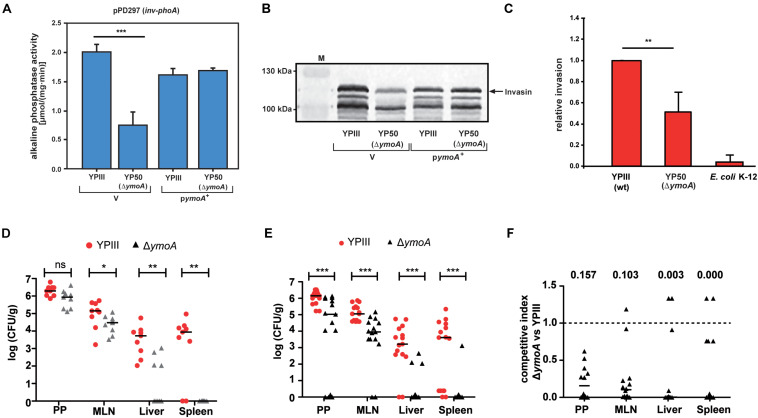
Influence of *ymoA* on the virulence of *Y. pseudotuberculosis*. *Y. pseudotuberculosis* wild-type strain YPIII and the mutant strain YP50 (Δ*ymoA*) harboring the *inv-phoA* fusion plasmid pPD297 and the empty vector control pACYC184 (V) or the *ymoA*+ plasmid pAKH71 were grown overnight in LB medium at 25°C. **(A)** Alkaline phosphatase activity from overnight cultures was determined and is given in μmol min^–1^ mg^–1^. The data represent the average SD from at least three different experiments each done in duplicate. The statistical significances between the wild-type and the *ymoA* mutant were determined by the ANOVA test. *P*-values: ***: <0.001. **(B)** In parallel, whole cell extracts of the strains were prepared and analyzed by Western blotting with a monoclonal antibody directed against invasin. A molecular weight marker is loaded on the left. **(C)** HEp-2 cells were infected with wild-type *Y. pseudotuberculosis*, Δ*ymoA Y. pseudotuberculosis* and *E. coli* K-12 and relative invasion was calculated. Each bar represents the average of three independent experiments relative to the invasion rate promoted by the *Y. pseudotuberculosis* wild-type strain, defined as 1.0. The statistical significances between the wild-type and the *ymoA* mutant were determined by the Students-test. *P*-values: **: <0.01. **(D)** BALB/c mice were infected intragastrically with an inoculum of 5⋅10^8^ CFU of *Y. pseudotuberculosis* wild-type YPIII and the *ymoA* mutant YP50. Colonization of Peyer’s patches (PP), mesenteric lymph nodes (MLN), liver and spleen after 3 days of infection is displayed. **(E)** For co-infection experiments, an inoculum with 1⋅10^9^ CFU of an equal mixture comprising *Y. pseudotuberculosis* wild-type YPIII and the *ymoA* mutant strain YP50 was infected into BALB/c mice via the orogastric route. After 3 days of infection, mice were sacrificed and the number of bacteria in homogenized host tissues and organs was determined by plating. Data are represented in scatter plots of numbers of CFU per gram as determined by counts of viable bacteria on plates. The statistical significances between the wild-type and the *ymoA* mutant were determined by the Mann–Whitney-test. *P*-values: *: <0.05; **: <0.01; ***: <0.001. **(F)** Data are graphed as competitive index values for the tissue samples from one mouse. Solid lines indicate median values. A competitive index score of 1 denotes no difference in the virulence compared to YPIII. Underlined values indicated the competitive index scores of the *ymoA* mutant relative to the wild-type.

Transcriptome analysis and subsequent investigation revealed that YmoA not only inhibits the expression of the pYV-encoded antiphagocytic T3SS-associated virulence factors important to resist innate immune cell attacks through, but also modulates the expression of multiple colonization factors crucial for the initial infection phase (Supplementary Table 4). In fact, absence of YmoA strongly reduced synthesis of the RovA-dependent internalization factor invasin (InvA) (Figures 9A,B) which decreased the ability of *Y. pseudotuberculosis* to invade human cells by 50% (Figure 9C). This suggested that YmoA acts as a key virulence modulator which supports reprogramming from the initial colonization into the host defense mode.

To further define the overall influence of *ymoA* on bacterial pathogenesis, we compared the ability to colonize lymphatic tissues and organs between the *Y. pseudotuberculosis* wild-type strain YPIII and an isogenic *ymoA*-deficient strain (YP50) in a mouse infection model. First, we examined success of the infection in BALB/c mice 3 days after oral uptake of 5 × 10^8^ bacteria by quantifying the number of bacteria that reached and survived in the Peyer’s patches (PP), the mesenterial lymph nodes (MLN), liver and spleen (Figure 9D). Significantly reduced numbers of the *ymoA* mutant bacteria were recovered from the MLNs and the organs. Attenuation of the *ymoA* mutant strain was even more evident when competition experiments were performed with 1 × 10^9^ bacteria in an inoculum of an equal mixture of the wild-type and the *ymoA*-deficient strain (Figure 9E). Ten times less bacteria of the mutant were identified in the PPs and the MLNs, and only few numbers of bacteria were able to reach liver and spleen. Calculations of the competitive index of the mutant relative to wild-type clearly indicated that presence of YmoA is advantageous for the colonization of *Y. pseudotuberculosis* in all tested organs (Figure 9F).

## Discussion

Rapid adaptation of the gene expression profile by bacterial pathogens to changing environments within their hosts is a prerequisite to persist and establish a successful infection. Complex regulatory networks including global transcriptional and post-transcriptional regulatory factors orchestrate global cellular changes and control expression of virulence factors in close association with stress adaptation and metabolic functions. The carbon storage regulator (Csr) system composed of the RNA-binding protein CsrA and at least two regulatory RNAs (CsrB and CsrC) play a central role in the control of many host-pathogen interactions (Lucchetti-Miganeh et al., 2008; Kusmierek and Dersch, 2017; Romeo and Babitzke, 2018; Pourciau et al., 2020). In this report, we show that the *Yersinia* modulating protein YmoA is crucial for the control of Csr-type regulatory RNAs and reprograms virulence-relevant functions important for different stages of the infection (Figure 1B).

**FIGURE 10:**
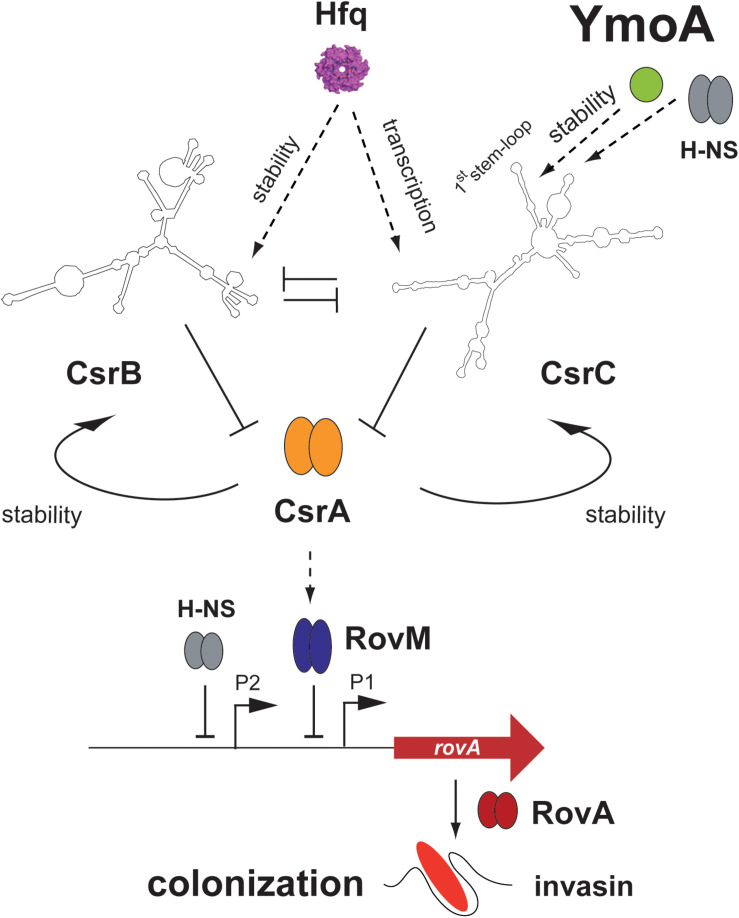
Influence of YmoA on the CsrABC-RovM-RovA-InvA cascade in *Y. pseudotuberculosis*. YmoA is a global virulence regulator of *Y. pseudotuberculosis* which is expressed under environmental conditions reflecting the initial stages of infection. It activates the synthesis of early-phase cell internalization factor invasin (InvA) which is important for colonization. This seems to occur together with H-NS, which is known to form heteromeric complexes with YmoA, through regulation of the degradation of the regulatory RNA CsrC at the first stem-loop structure of the CsrC RNA. Influence of YmoA on CsrC occurs independently of Hfq, which was also involved in Csr system control. YmoA and H-NS influence on CsrC is indirect and most likely occurs through RNA-processing factors. As a consequence of YmoA-mediated CsrC upregulation the CsrA-RovM-RovA regulator cascade is activated, leading to expression of colonization factors such as invasin. Dark arrows display direct activation, T represents repression, and dashed arrows indicate (indirect) activation by an unknown molecular mechanism.

The YmoA protein of *Yersinia* belongs to a class of small proteins which modulate virulence gene expression in *Enterobacteriaceae* in response to environmental changes, e.g., temperature (Madrid et al., 2007). YmoA has a high amino acid sequence identity to and a similar overall fold consisting of four helices to Hha of *E. coli* which was identified as repressor of α-hemolysin production and other virulence genes (Mourino et al., 1996; Sharma and Zuerner, 2004; McFeeters et al., 2007). Also, the *Yersinia* YmoA protein is a key regulator of pathogenicity factors. It was identified as a thermosensitive transcriptional silencer for *lcrF* expression. LcrF is a key regulator for the pYV-encoded temperature-induced T3SS and *yop* effector genes important to prevent the immune defense after entry of the gut-associated lymphatic tissues (Mikulskis et al., 1994; Jackson et al., 2004; Böhme et al., 2012). Hence, by controlling *lcrF* expression, YmoA regulates one of the most crucial pathogenicity factors for infection of the human host. In this study, we show that YmoA also activates expression of virulence genes important for host tissue colonization during early stages of the infection. YmoA induces expression of the virulence regulator gene *rovA* and the RovA-dependent *invA* gene by downregulation of the LysR repressor RovM, and this is mediated through YmoA-dependent changes of the global post-transcriptional Csr system (Figure 10). YmoA was found to enhance the stability of the CsrC RNA and this was shown to involve sequences within the 5′-region of the CsrC RNA predicted to form an extended stem-loop.

Turnover of regulatory RNAs is a highly regulated process. Potential pathways for modulation include recruitment or inhibition of RNA decay enzymes (RNases), formation of stabilizing RNA secondary structures and/or alteration of the expression, activity or localization of RNA-binding factors. In *E. coli* and *Salmonella enterica* serovar Typhimurium, CsrB and CsrC decay involves the essential enzyme RNase E, a single-strand-specific endoribonuclease, which associates with the 3′–5′ processive exonuclease polynucleotide phosphorylase (PNPase), the glycolytic enzyme enolase, and an RNA helicase (RhlB/CsdA) to form an RNA-degrading complex (degradosome) (Suzuki et al., 2006; Viegas et al., 2007). Processing of the *Salmonella* CsrC RNA at one of the longer 3′ stems is also RNase III-dependent (Viegas et al., 2007). Here, we show that a 5′-terminal segment between nt +24 and +57 of the *Yersinia* CsrC RNA which is able to form a stem-loop structure is important for its stabilization. It is likely that the accessibility of potential ribonuclease cleavage sites is inhibited by this structured RNA element. However, none of the genes for RNase III (*rnc*), PNPase (*pnp*), and RNase E (*rne*) were differently expressed in the Δ*ymoA* mutant (Supplementary Table 4). As this part of the RNA varies significantly from *E. coli* and *Salmonella* CsrC and no smaller CsrC transcripts were detectable, it seems that CsrC decay mechanism in *Yersinia* differs from the other CsrC RNAs.

As absence of the hairpin sequence in the *csrC* gene eliminates YmoA influence on CsrC stabilization, it is also possible that YmoA controls RNA-binding proteins, protecting CsrC from decay. Although the abundance of CsrB and CsrC of *Y. pseudotuberculosis* is positively affected by the RNA chaperone Hfq, we show that YmoA acts independently of Hfq. As Hfq is still able to induce *csrC*(+4)-*lacZ* fusion, it is more likely that Hfq activates *csrC* expression indirectly through control of (an)other sensory or regulatory RNA(s). We further found that also the RNA-binding protein CsrA is crucial for CsrC abundance in *Yersinia*, and absence of YmoA could be partly overcome by CsrA overexpression. This implies that stabilization of CsrC by CsrA might either impede the activity of a YmoA-dependent destabilizing factor or CsrA might act as a stabilization factor itself. The most highly conserved element of CsrA binding sites is a GGA sequence. Five GGA motifs are located within the stabilizing 5′ region of the CsrC RNA, one in the loop, two at the 5′- and 3′-end of the hairpin structure and one at the start and end of the sequence (Figure 4C), which could assist in the formation of the stabilizing RNA element and/or protection of RNase cleavage sites. In this case, sequestration of CsrA to other target sequences would destabilze CsrC. Although overall CsrA levels remain unchanged in the absence of YmoA (Figure 3B), it is possible that global transcriptional changes of 289 genes detected in the Δ*ymoA* mutant – the great majority (79%) of which were upregulated – are associated with a significant increase of alternative CsrA target transcripts, which would reduce the availability of free CsrA to bind and stabilize the CsrC transcript. Similarly, CsrB and CsrC synthesis in *E. coli* is positively regulated by CsrA, but their stability was essentially identical between wild-type and the *csrA* mutant. This indicated that CsrA does not directly affect CsrB and CsrC RNA degradation in this organism (Gudapaty et al., 2001; Weilbacher et al., 2003).

Several years ago, Saxena and Gowrishankar (2011) showed that overexpression of the YmoA/Hha paralog YdgT suppressed the phenotypes of *rho* and *nusG* mutants. They suggest a model whereby YdgT modulates Rho-dependent transcription termination, by compensating for Rho loss (Saxena and Gowrishankar, 2011). Following the *E. coli* model, YmoA would increase transcriptional termination, what is not observed with *csrC*. Nonetheless, the hairpin structure of CsrC could be involved in premature transcriptional termination which could be inhibited by YmoA. However, no short, truncated CsrC transcripts have been identified with CsrC-specific probes.

Several studies further indicate that the global YmoA/Hha modulators exert their effect through heterocomplex formation with H-NS (Nieto et al., 2002; Madrid et al., 2007). As YmoA was also found to be structurally similar to H-NS, it was further suggested that YmoA may intercalate into higher-order structures by substituting for an H-NS dimer (McFeeters et al., 2007). H-NS is a highly abundant DNA-binding protein, which binds and polymerizes along AT-rich, curved DNA sites and acts as global regulator of gene expression in response to environmental signals (Bouffartigues et al., 2007; Fang and Rimsky, 2008). Heterocomplex formation with YmoA modifies the biological activity of H-NS and reduces or increases its ability to repress transcription (Nieto et al., 2002; Banos et al., 2008). Alike YmoA, H-NS controls the amount of CsrC and complements the decrease of CsrC levels in an *ymoA* mutant, indicating that YmoA and H-NS most likely acts in a cooperative manner to modulate transcription of factors controlling CsrC decay.

More recently, YmoA/Hha, together with YmoB/TomB(YbaJ) encoded in the same operon, was found to constitute a type II toxin-antitoxin (TA), which is implicated in the formation of persister cells and biofilms by repressing type I fimbriae expression in related *Enterobacteriaceae* (García-Contreras et al., 2008; Marimon et al., 2016). In *E. coli*, TomB(YbaJ) transiently interacts with the YmoA homolog Hha and initiates spontaneous oxidation of a conserved Cys residue leading to a destabilization of Hha. Its function can be replaced by the TomB homolog YmoB of *Yersinia* (Marimon et al., 2016). Interestingly, the abundance of the *ymoB* transcript is strongly (81-fold) increased in the absence of *ymoA* (Supplementary Table 4), indicating a negative autoregulatory loop. This reveals also the possibility that enhanced degradation of CsrC and changes of RovM, RovA and InvA levels in the Δ*ymoA* mutant could be promoted through upregulation of YmoB. The overall contribution of the TA system is not yet fully understood, but induction of the TA loci by environmental stress conditions led to the idea that these systems enhance bacterial fitness under adverse conditions and play roles in pathogenesis (Norton and Mulvey, 2012). Our work showing that YmoA manipulates the global post-transcriptional Csr system, influences multiple fitness- and virulence-relevant components and has a strong effect on virulence, supports this assumption.

Our transcriptome analysis revealed a global impact of YmoA on early and later stage virulence genes. This includes virulence traits important for host tissue colonization such as cell adhesion and invasion factors (invasin, Ail, PsaA adhesin, and fimbriae), toxins and type VI secretion system components as well as immune cell defense strategies (T3SS-Yops). Moreover, a variety of genes for stress responses experienced in the initial stage of the infection (oxidative stress, starvation, and pH) are activated by YmoA, whereas factors promoting translation and proper folding of proteins (e.g., heat stress/shock genes) are repressed. In addition, metabolic activities related to the amino acid/nitrogen metabolism and assimilation are activated, whereas the metabolism of many carbon sources is repressed by YmoA (Figures 1B,C and Supplementary Table 4). This reprogramming implicates control of the Csr system which (i) plays a central role coordinating virulence gene expression with adaptative responses functions for different nutritional demands and stress resistance during infections (Lucchetti-Miganeh et al., 2008; Timmermans and Van Melderen, 2010; Bücker et al., 2014; Kusmierek and Dersch, 2017) and (ii) promotes transition between different physiological stages of pathogens, e.g., the switch from an acute to a chronic infection or from a transmission into an intracellular replication state (Molofsky and Swanson, 2003; Goodman et al., 2004; Laskowski and Kazmierczak, 2006; Mulcahy et al., 2008; Brencic and Lory, 2009; Sahr et al., 2009).

One important environmental parameter, which induce global alterations in the gene expression and reprogramming between the different virulence-related stages of *Yersinia*, is temperature (Nuss et al., 2015; Schmühl et al., 2019). As YmoA turnover by the ClpP and Lon proteases is modulated by temperature (Jackson et al., 2004) (Supplementary Figure 8), it is tempting to speculate that YmoA plays a crucial role adjusting the Csr regulon in response to temperature which is pivotal for the establishment of a successful infection.

Based on our analysis we assume that thermosensitive YmoA promotes expression of very early-stage virulence-relevant traits to promote initiation of the infection. Subsequent degradation of YmoA at 37°C within the host will then trigger an upregulation of pathogenicity factors, i.e., toxin CNF_Y_ and the antiphagocytic T3SS/Yop machinery, and improve use of carbon sources, which are crucial for the establishment of the infection in the intestine and underlying lymphoid tissues. Co-induction of mechanisms that promote protein translation and folding may guarantee a rapid and highly efficient production of these virulence traits. This global impact of YmoA on virulence was visible in non-competitive mouse infection studies and was even more noticeable in competitive assays in which the *ymoA* mutant was clearly outcompeted by the wild-type. Many questions concerning the molecular interactions between YmoA and the Csr system, and significance of YmoA-controlled genes for virulence remain to be solved. Yet, it is foreseeable that the YmoA-Csr control network is a valuable target for antibacterial therapeutics to block the bacteria at specific stages during infection.

## Data Availability Statement

The datasets generated for this study can be found in online repositories. The names of the repository/repositories and accession number(s) can be found in the article/Supplementary Material.

## Ethics Statement

The animal study was reviewed and approved by Niedersächsisches Landesamt für Verbraucherschutz und Lebensmittelsicherheit: animal licensing committee permission no. 33.9.42502-04-055/09.

## Author Contributions

AH, KB, and PD designed the research. AH, KB, and SL performed the experiments. AH, KB, SL, PD, ASS, and YG analyzed the data. AH, KB, and PD interpreted the data. AH and PD wrote the manuscript. AH and ASS edited the manuscript. PD acquired funding. All authors contributed to the article and approved the submitted version.

## Conflict of Interest

The authors declare that the research was conducted in the absence of any commercial or financial relationships that could be construed as a potential conflict of interest.

## Publisher’s Note

All claims expressed in this article are solely those of the authors and do not necessarily represent those of their affiliated organizations, or those of the publisher, the editors and the reviewers. Any product that may be evaluated in this article, or claim that may be made by its manufacturer, is not guaranteed or endorsed by the publisher.

## Footnotes

1
http://www.genomics.agilent.com

2
www.r-project.org

3
www.bioconductor.org

## Supplementary Material

Supplementary Figure 1 Influence of the growth medium, growth phase and temperature on YmoA synthesis. *Y. pseudotuberculosis* strain YPIII was grown in LB or minimal medium A (MMA) to exponential (OD_600_ = 0.8) or stationary phase at 25 and 37°C. Whole cell extracts of the cultures were prepared, proteins were separated on 18% TRICINE polyacrylamide gels and analyzed by Western blotting with a polyclonal antibody directed against YmoA and CsrA. Cell extracts of an *ymoA* and a *csrA* mutant strain were used as controls. A used prestained molecular weight marker is indicated on the left. Unspecifically detected proteins (c) were used as loading control.

Supplementary Figure 2 YmoA has no influence on *rovA* expression through H-NS. *Y. pseudotuberculosis* strain YPIII and YP50 (Δ*ymoA*) harboring the empty vector (V), or a dominant negative *hns*^∗^ allele (pAKH31) were cultivated overnight in LB medium at 25°C. Whole cell extracts were prepared, separated by SDS-PAGE and analyzed by Western blotting with a polyclonal antibody directed against RovA. Cell extracts of a *rovA* mutant was used as control. Unspecifically detected proteins (c) were used as loading control.

Supplementary Figure 3 Influence of YmoA on the Csr system occurs through CsrC. *Y. pseudotuberculosis* strains YPIII (wild-type), YP50 (Δ*ymoA*), YP69 (Δ*csrB*), and YP75 (Δ*csrB* and Δ*ymoA*) were grown in LB at 25°C overnight. **(A)** Total RNA of the strains was extracted, and CsrC transcripts were detected by Northern blotting with a CsrC-specific probe. The 16S rRNAs are shown as RNA loading control. **(B)** In parallel, whole-cell extracts were prepared from the cultures and analyzed by Western blotting with a polyclonal antibody directed against RovM. A molecular weight marker is loaded on the left. Unspecifically detected proteins (c) were used as loading control.

Supplementary Figure 4 YmoA has a positive effect on CsrC stability. **(A)** *Y. pseudotuberculosis* strains YPIII (wild-type) and YP50 (Δ*ymoA*) harboring a P*_*tet*_*:*lacZ* expression construct (pTT1) were grown to exponential phase in minimal medium at 25°C in the presence of 0.5 nM anhydrotetracycline. β-galactosidase activity from the overnight cultures was determined and is given in μmol min^–1^ mg^–1^ for comparison. The data represent the average ± SD from at least three different experiments each done in duplicate. **(B)** *Y*. *pseudotuberculosis* strains YPIII (wild-type) and YP50 (Δ*ymoA*) harboring the empty vector (pHSG576) or a P*_*tet*_*:*csrC* expression construct (pKB47) were grown to exponential phase in minimal medium at 25°C in the presence of 0.5 nM anhydrotetracycline. Total RNA of the strains was extracted and CsrC was detected by Northern blotting with a CsrC-specific probe. The 16S and 23S rRNAs are shown as RNA loading control.

Supplementary Figure 5 Hfq influence on RovA and RovM levels. *Y. pseudotuberculosis* strain YPIII and YP80 (Δ*hfq*) carrying the vector pAKH85 (pV) or its *hfq*^+^ (pAKH115) were grown in LB at 25°C overnight. Whole-cell extracts from the overnight cultures were prepared and analyzed by Western blotting with polyclonal antibodies directed against RovA or RovM. Unspecifically detected proteins (c) were used as loading control.

Supplementary Figure 6 YmoA influence on Hfq production. *Y. pseudotuberculosis* strain YPIII and YP50 (Δ*ymoA*) carrying the vector pAKH85 (pV) or its *ymoA*^+^ derivative (pAKH71) were grown in LB at 25°C overnight. Whole-cell extracts from the overnight cultures were prepared and analyzed by Western blotting with a polyclonal antibody directed against Hfq. An unspecifically detected protein (c) was used as loading control.

Supplementary Figure 7 Analysis of YmoA and H-NS binding to CsrC and *yscW.* **(A)** CsrC RNA (+1 to +150) and a 5S RNA fragment serving as a negative control as well as **(B)** the *yscW-lcrF* promoter fragment and a *csiD* fragment of *E. coli* serving as negative control were incubated without or with increasing amounts of purified *Y. pseudotuberculosis* YmoA in the presence or absence of H-NS or H-NS alone. The samples were separated on 8% polyacrylamide gels. The positions of the gene fragments are indicated, and the formation of higher molecular complexes is shown by an arrow.

Supplementary Figure 8 Temperature-dependent YmoA degradation. Whole cell extracts from overnight cultures of *Y. pseudotuberculosis* strains YPIII (wild-type), YP63 (Δ*clpP*), YP64 (Δ*lon*) and YP67 (Δ*clpP* Δ*lon*) grown in LB at 25 and 37°C were prepared, separated by 20% TRICINE-PAGE and analyzed by Western blotting using a polyclonal anti-YmoA antibody. Whole cell extract of an overnight culture of YP50 (Δ*ymoA*) was used as negative control. A molecular weight marker was loaded on the left, the YmoA protein is indicated by an arrow. Unspecifically detected proteins (c) were used as loading control.
