# Supplementary figures and images for: The Small Protein YmoA Controls the Csr System and Adjusts Expression of Virulence-Relevant Traits of Yersinia pseudotuberculosis
Source: Front Microbiol. 2021 Aug 3;12:706934. doi: 10.3389/fmicb.2021.706934 (PMC8369931; doi:10.3389/fmicb.2021.706934)

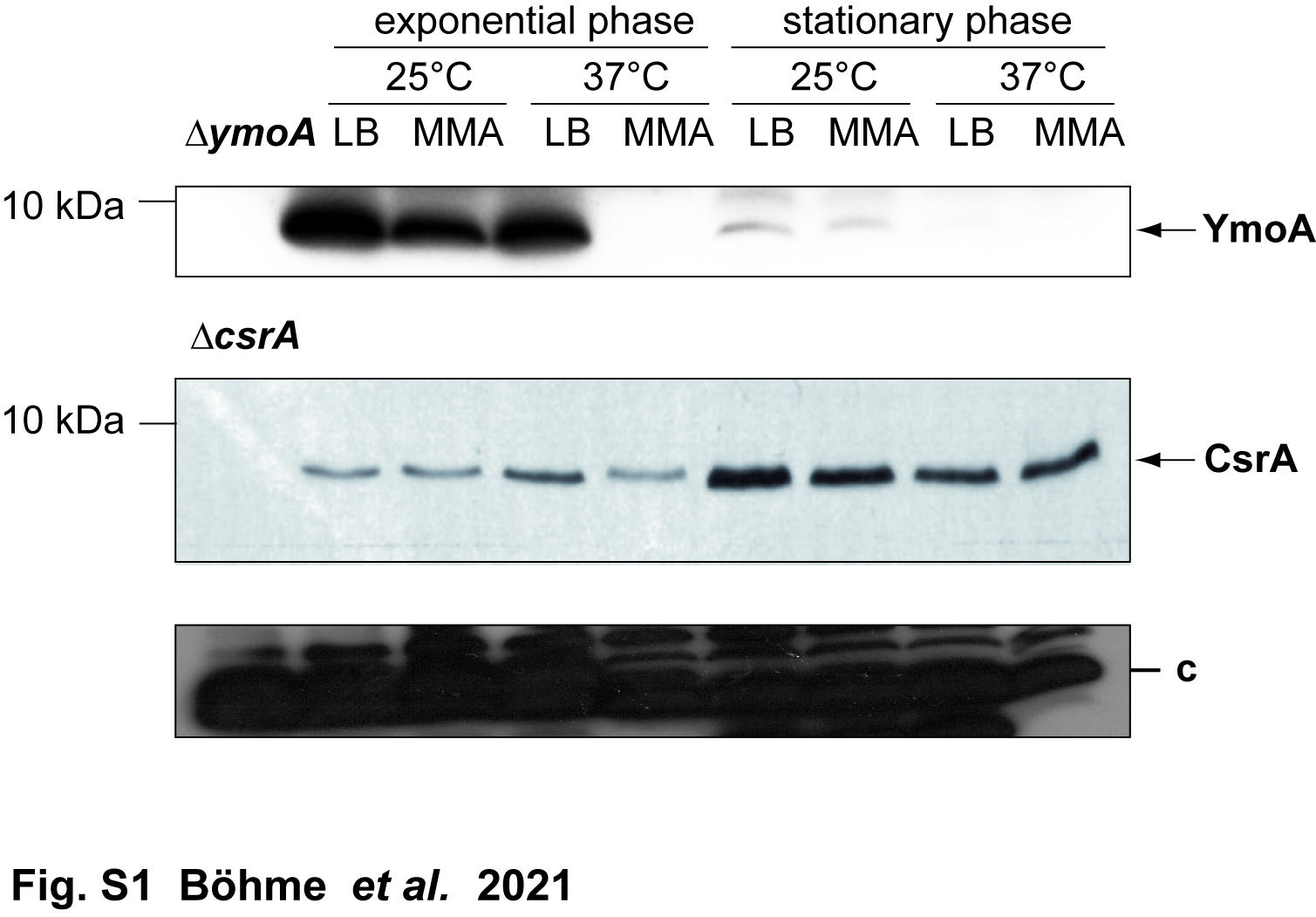

Supplement: Supplementary Figure 1 — Influence of the growth medium, growth phase and temperature on YmoA synthesis. Y. pseudotuberculosis strain YPIII was grown in LB or minimal medium A (MMA) to exponential (OD600 = 0.8) or stationary phase at 25 and 37°C. Whole cell extracts of the cultures were prepared, proteins were separated on 18% TRICINE polyacrylamide gels and analyzed by Western blotting with a polyclonal antibody directed against YmoA and CsrA. Cell extracts of an ymoA and a csrA mutant strain were used as controls. A used prestained molecular weight marker is indicated on the left. Unspecifically detected proteins (c) were used as loading control. [file Image_1.TIF]

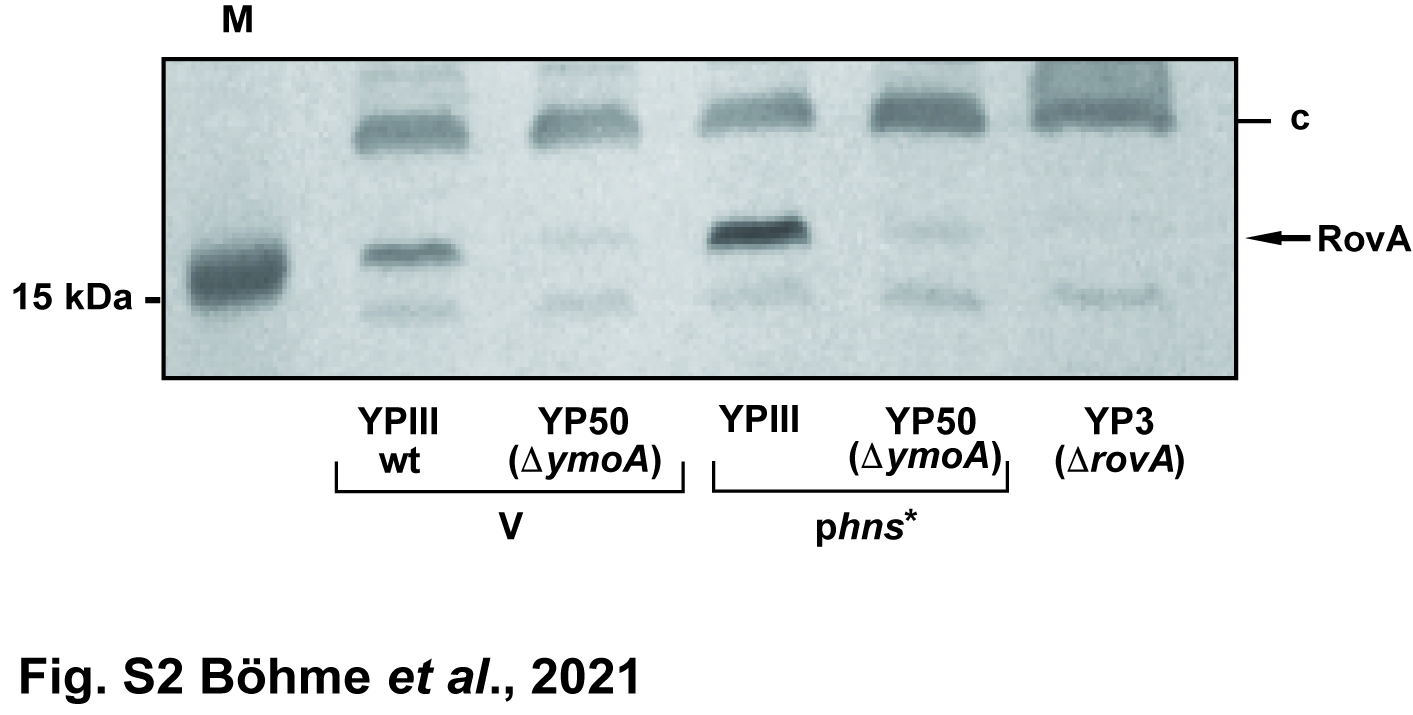

Supplement: Supplementary Figure 2 — YmoA has no influence on rovA expression through H-NS. Y. pseudotuberculosis strain YPIII and YP50 (ΔymoA) harboring the empty vector (V), or a dominant negative hns∗ allele (pAKH31) were cultivated overnight in LB medium at 25°C. Whole cell extracts were prepared, separated by SDS-PAGE and analyzed by Western blotting with a polyclonal antibody directed against RovA. Cell extracts of a rovA mutant was used as control. Unspecifically detected proteins (c) were used as loading control. [file Image_2.TIF]

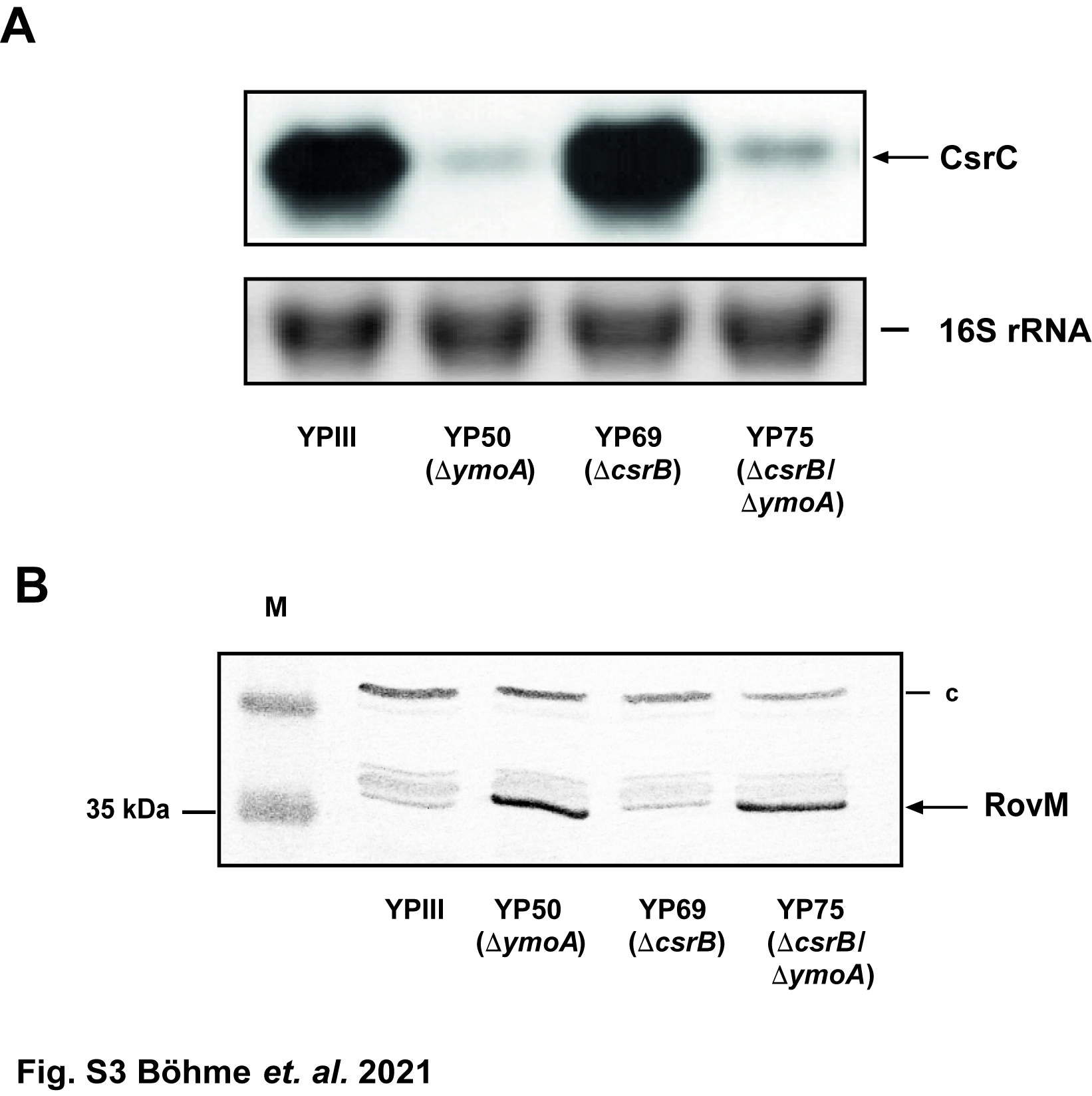

Supplement: Supplementary Figure 3 — Influence of YmoA on the Csr system occurs through CsrC. Y. pseudotuberculosis strains YPIII (wild-type), YP50 (ΔymoA), YP69 (ΔcsrB), and YP75 (ΔcsrB and ΔymoA) were grown in LB at 25°C overnight. (A) Total RNA of the strains was extracted, and CsrC transcripts were detected by Northern blotting with a CsrC-specific probe. The 16S rRNAs are shown as RNA loading control. (B) In parallel, whole-cell extracts were prepared from the cultures and analyzed by Western blotting with a polyclonal antibody directed against RovM. A molecular weight marker is loaded on the left. Unspecifically detected proteins (c) were used as loading control. [file Image_3.TIF]

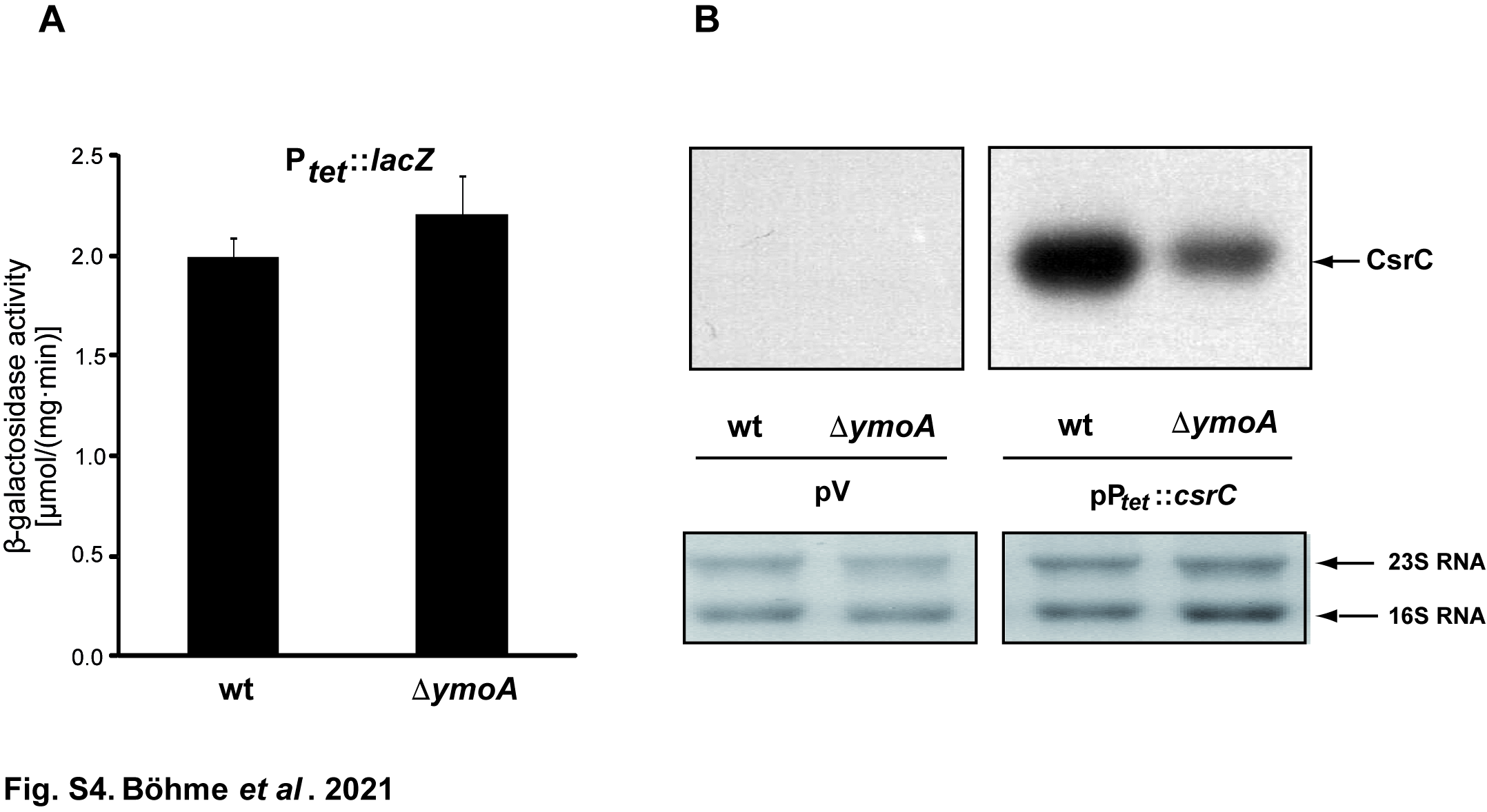

Supplement: Supplementary Figure 4 — YmoA has a positive effect on CsrC stability. (A) Y. pseudotuberculosis strains YPIII (wild-type) and YP50 (ΔymoA) harboring a Ptet:lacZ expression construct (pTT1) were grown to exponential phase in minimal medium at 25°C in the presence of 0.5 nM anhydrotetracycline. β-galactosidase activity from the overnight cultures was determined and is given in μmol min–1 mg–1 for comparison. The data represent the average ± SD from at least three different experiments each done in duplicate. (B) Y. pseudotuberculosis strains YPIII (wild-type) and YP50 (ΔymoA) harboring the empty vector (pHSG576) or a Ptet:csrC expression construct (pKB47) were grown to exponential phase in minimal medium at 25°C in the presence of 0.5 nM anhydrotetracycline. Total RNA of the strains was extracted and CsrC was detected by Northern blotting with a CsrC-specific probe. The 16S and 23S rRNAs are shown as RNA loading control. [file Image_4.TIF]

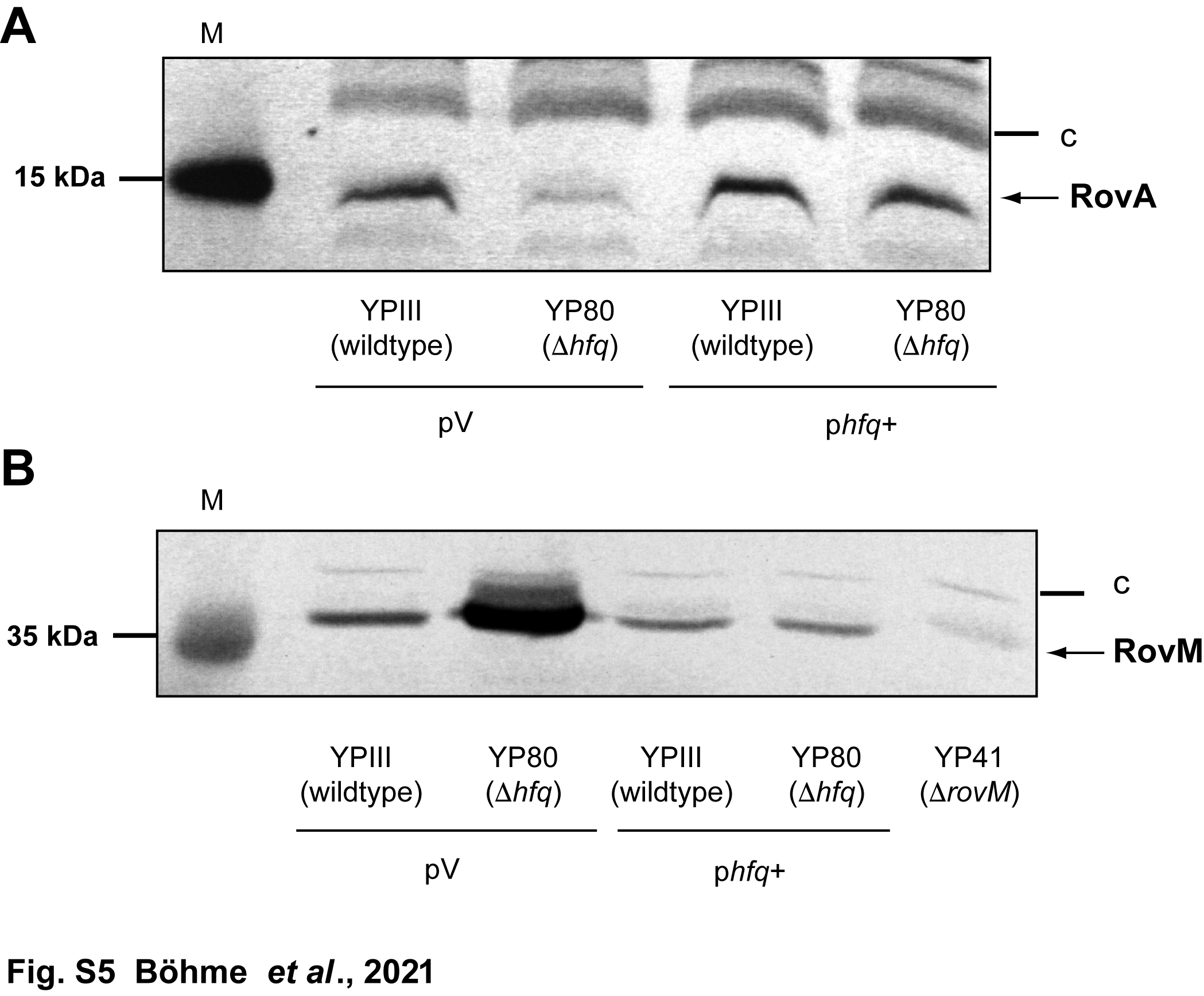

Supplement: Supplementary Figure 5 — Hfq influence on RovA and RovM levels. Y. pseudotuberculosis strain YPIII and YP80 (Δhfq) carrying the vector pAKH85 (pV) or its hfq+ (pAKH115) were grown in LB at 25°C overnight. Whole-cell extracts from the overnight cultures were prepared and analyzed by Western blotting with polyclonal antibodies directed against RovA or RovM. Unspecifically detected proteins (c) were used as loading control. [file Image_5.TIF]

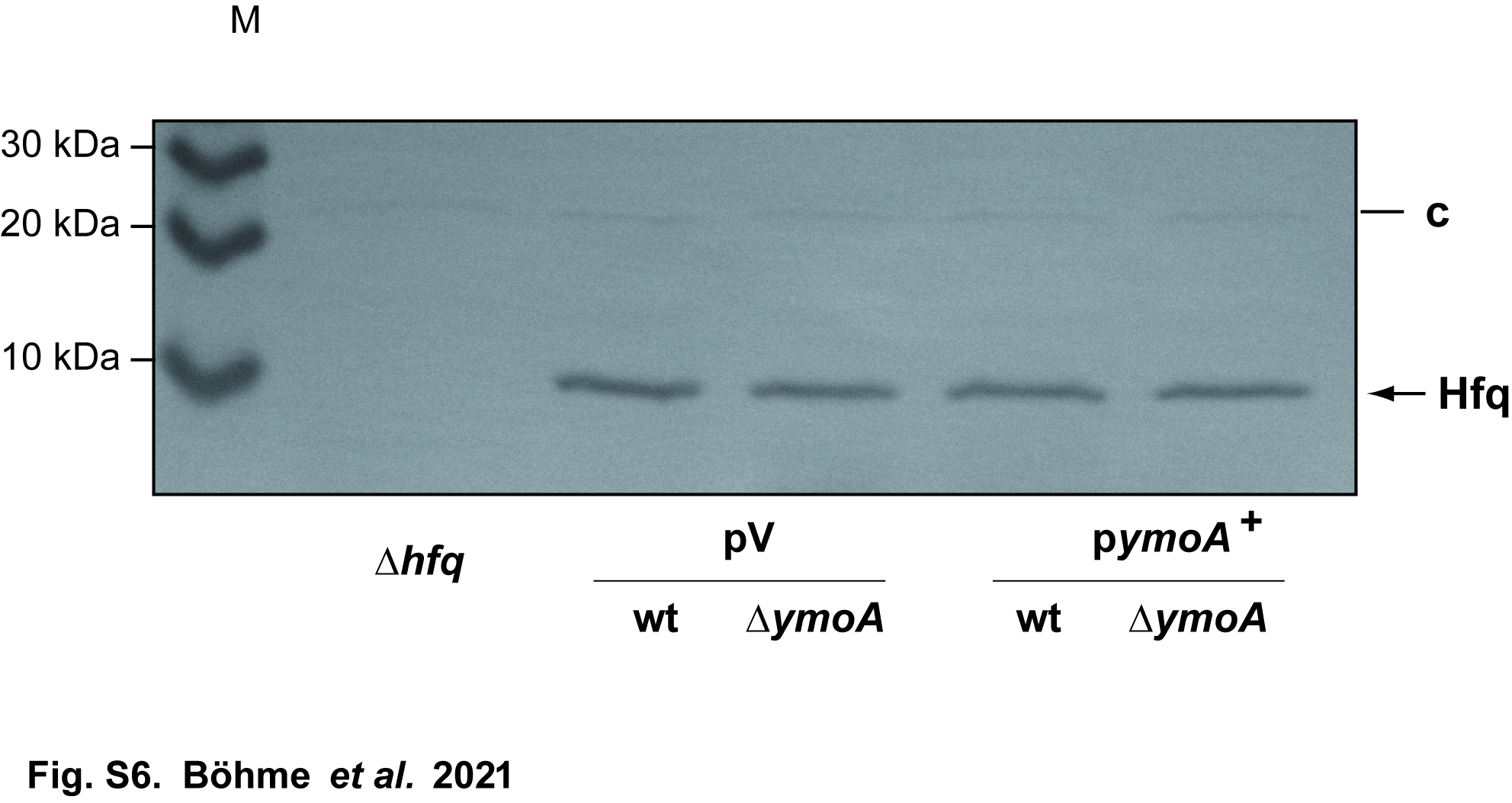

Supplement: Supplementary Figure 6 — YmoA influence on Hfq production. Y. pseudotuberculosis strain YPIII and YP50 (ΔymoA) carrying the vector pAKH85 (pV) or its ymoA+ derivative (pAKH71) were grown in LB at 25°C overnight. Whole-cell extracts from the overnight cultures were prepared and analyzed by Western blotting with a polyclonal antibody directed against Hfq. An unspecifically detected protein (c) was used as loading control. [file Image_6.TIF]

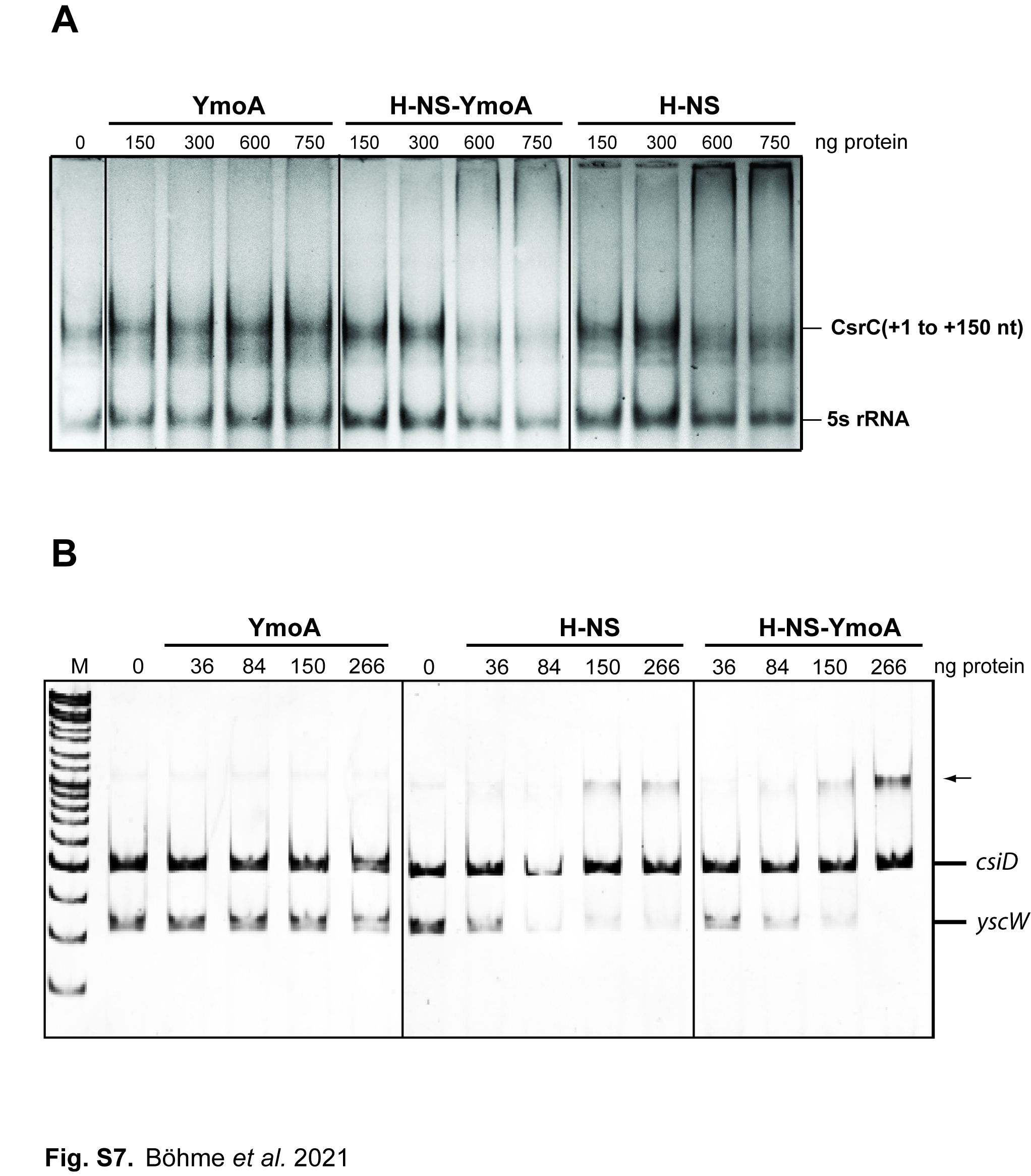

Supplement: Supplementary Figure 7 — Analysis of YmoA and H-NS binding to CsrC and yscW. (A) CsrC RNA (+1 to +150) and a 5S RNA fragment serving as a negative control as well as (B) the yscW-lcrF promoter fragment and a csiD fragment of E. coli serving as negative control were incubated without or with increasing amounts of purified Y. pseudotuberculosis YmoA in the presence or absence of H-NS or H-NS alone. The samples were separated on 8% polyacrylamide gels. The positions of the gene fragments are indicated, and the formation of higher molecular complexes is shown by an arrow. [file Image_7.TIF]

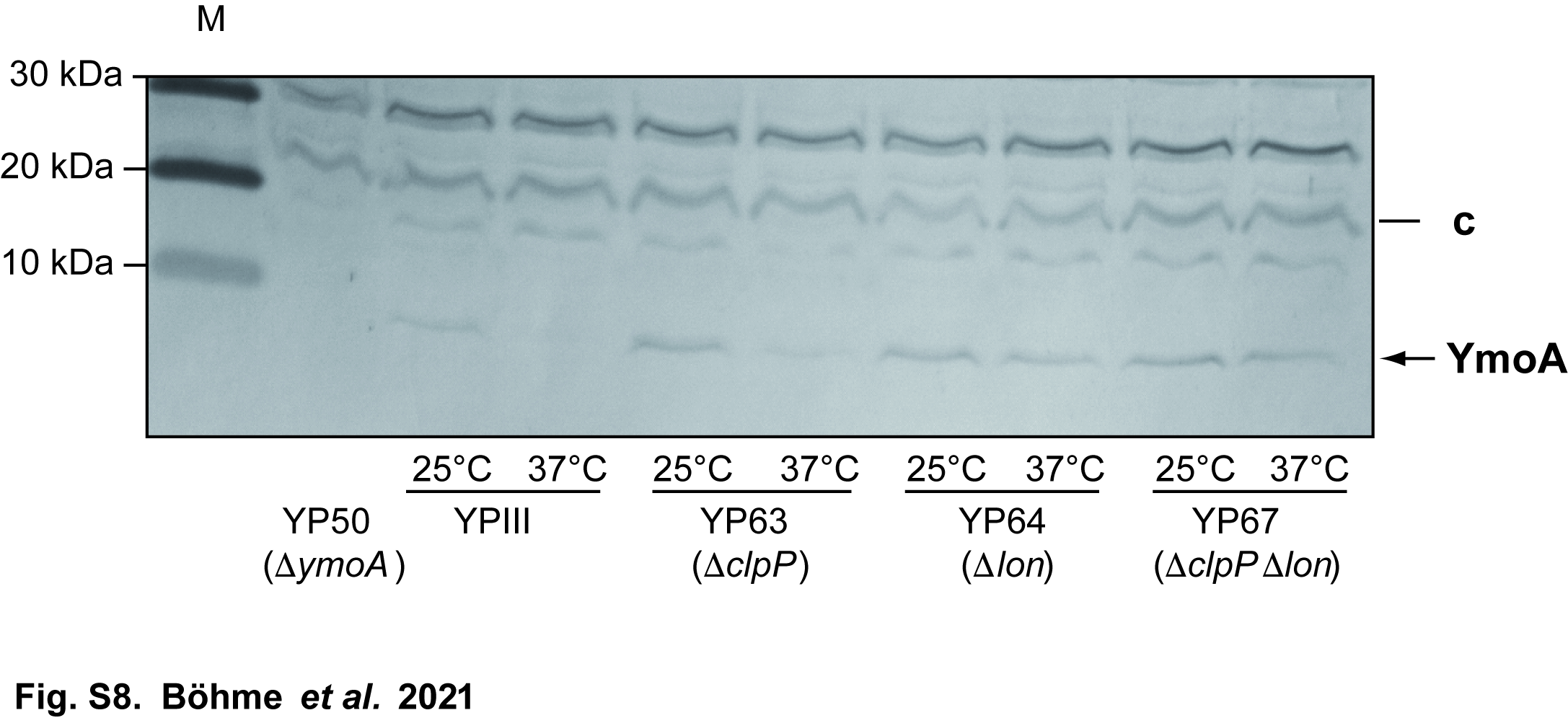

Supplement: Supplementary Figure 8 — Temperature-dependent YmoA degradation. Whole cell extracts from overnight cultures of Y. pseudotuberculosis strains YPIII (wild-type), YP63 (ΔclpP), YP64 (Δlon) and YP67 (ΔclpP Δlon) grown in LB at 25 and 37°C were prepared, separated by 20% TRICINE-PAGE and analyzed by Western blotting using a polyclonal anti-YmoA antibody. Whole cell extract of an overnight culture of YP50 (ΔymoA) was used as negative control. A molecular weight marker was loaded on the left, the YmoA protein is indicated by an arrow. Unspecifically detected proteins (c) were used as loading control. [file Image_8.TIF]
