# Supplementary material for: The Small Protein YmoA Controls the Csr System and Adjusts Expression of Virulence-Relevant Traits of Yersinia pseudotuberculosis
Source: Front Microbiol. 2021 Aug 3;12:706934. doi: 10.3389/fmicb.2021.706934 (PMC8369931; doi:10.3389/fmicb.2021.706934)
Supplement: Supplementary file 9 [file Table_1.DOCX]

**Table S1.** Bacterial strains and plasmids.

Strains, Plasmids Description Source and reference

Bacterial strains

*E. coli* K-12
CC118λpir F^-^ ∆(*ara-leu*)7697 ∆(*lacZ*)74 ∆(*phoA*)20 *araD139* ([Manoil & Beckwith, 1986](#_ENREF_47))
 *galE galK thi rpsE rpoB arfE*^am^ *recA1*, λ*pir*

BL21 λDE3 F^-^ *ompT gal dcm lon hsdSB* (r_B_^-^ m_B_^-^) λDE3 ([Studier & Moffatt, 1986](#_ENREF_80))

KB4 BL21λDE3 (Δ*stpA,* Δ*hns,* Δ*hha*) ([Böhme *et al*., 2012](#_ENREF_5))

*Y. pseudotuberculosis*

YPIII pIB1, wild type ([Bolin *et al.*, 1982](#_ENREF_6))

YP3 pIB1, *rovA*::Tn*10*(60)^a^; Cm^R^ ([Nagel *et al*., 2001](#_ENREF_62))

YP38 pIB1, *rovA-lacZ*(129)^b^, Ap^R^ (Heroven and Dersch, 2006)

YP41 pIB1, Δ*rovM*; Kn^R^ (Heroven and Dersch, 2006)

YP48 pIB1, Δ*csrC*; Kn^R^ ([Heroven *et al*., 2008](#_ENREF_30))

YP50 pIB1, Δ*ymoA*; Kn^R^ ([Böhme *et al*., 2012](#_ENREF_5))

YP51 pIB1, Δ*csrB*; Ap^R^ ([Heroven *et al*., 2008](#_ENREF_30))

YP53 pIB1, Δ*csrA*; Kn^R^ ([Heroven *et al*., 2008](#_ENREF_30))

YP63 pIB1, Δ*clpP*; Kn^R^ ([Herbst *et al*., 2009](#_ENREF_29))

YP64 pIB1, Δ*lon*; Ap^R^ ([Herbst *et al*., 2009](#_ENREF_29))

YP67 pIB1, Δ*lon,* Δ*clpP*; Kn^R^, Ap^R^ ([Herbst *et al*., 2009](#_ENREF_29))

YP69 pIB1, Δ*csrB* ([Heroven *et al*., 2008](#_ENREF_30))

YP73 pIB1, Δ*ymoA*; Δ*rovM*, Kn^R^ this study

YP72 pIB1, Δ*rovM* (Heroven *et al*. 2012)

YP75 pIB1, Δ*csrB,*  Δ*ymoA*; Kn^R^ this study

YP80 pIB1, Δ*hfq* (Heroven *et al*. 2012)

Plasmids

pACYC184 cloning vector, p15A, Cm^R^, Tet^R^ ([Chang & Cohen, 1978](#_ENREF_12))

pAKH11 pET28, *hns*^+^, Kn^R^ (Heroven *et al.,* 2004)

pAKH31 pK18, *hns**, Tet^R^ (Heroven and Dersch*,* 2006)

pAKH32 pK18, Tet^R^ (Heroven and Dersch, 2006)

pAKH42 pACYC184, *rovM*^+^, Cm^R^ (Heroven and Dersch, 2006)

pAKH47 pGP20, *rovA-lacZ*(17)^b^, Tet^R^ (Heroven and Dersch, 2006)

pAKH63 pGP20, *rovM-lacZ*(41)^b^, Tet^R^ (Heroven and Dersch, 2006)

pAKH71 pACYC184, *ymoA*^+^, Cm^R^ ([Böhme *et al*., 2012](#_ENREF_5))

pAKH74 pACYC184, *hns*^+^, Cm^R^ (Heroven and Dersch, 2006)

pAKH76 pHT124, *csrC-lacZ*(81)^b^ this study

pAKH77 pASK-IBA5plus, *ymoA*^+^, Ap^R^ ([Böhme *et al*., 2012](#_ENREF_5))

pAKH85 pACYC184, Cm^R^, Tet^S^ ([Heroven *et a*l., 2008](#_ENREF_30))

pAKH97 pHT124, *csrC-lacZ*(4)^c^, Ap^R^ ([Heroven *et al*., 2008](#_ENREF_30))

pAKH101 pHT124, *csrB-lacZ*(4)^c^, Ap^R^ ([Heroven *et al*., 2008](#_ENREF_30))

pAKH103 pHT124, *csrC-lacZ*(4)^c^, Ap^R^ (Nuss *et al*. 2014)

pAKH104 pHT124, *csrC-lacZ*(254)^c^, Ap^R^ this study

pAKH106 pHT124, *csrC-lacZ*(39)^c^, Ap^R^ this study

pAKH107 pHT124, *csrC-lacZ*(61)^c^, Ap^R^ this study

pAKH115 pACYC184, *hfq*^+^, Cm^R^ this study

pAKH119 pHSG575, *hfq*^+^, Cm^R^ this study

pBR322 cloning vector, Tet^R^, Ap^R^ Bolivar *et al*. (1977)

pGP20 protein fusion vector, ´*lacZ*, Tet^R^ P. Gerlach

pHSG575 pSC101, cloning vector, Cm^R^ ([Takeshita *et al.*, 1987](#_ENREF_82))

pHSG576 pSC101, cloning vector, Cm^R^ ([Takeshita *et al*., 1987](#_ENREF_82))

pHT124 promoter-probe vector, *lacZ*^+^, Ap^R^ ([Heroven *et al*., 2008](#_ENREF_30))

pKB4 pHSG575, *ymoA*^+^, Cm^R^ this study

pKB17 pHT124, *csrC*(Δ24-57)^d^*-lacZ*(81)^c^, Ap^R^ this study

pKB20 pHT124, *csrC-lacZ*(71)^c^, Ap^R^ this study

pKB47 pHSG575, P*_tet_*::*csrC, Cm*^R^ this study

pKB49 pHSG575, P*_csrC_*::*csrC*(Δ24-57)^d^*, Cm*^R^ this study

pKB59 pHSG576, P*_csrC_*::*csrC, Cm*^R^ this study

pKB60 pHSG576, *csrA*^+^, Cm^R^ (Kusmierek *et al.* 2019)

pKB63 pTS02, *csrA*’-‘*lacZ*, Ap^R^ (Böhme *et al*. 2012)

pKOBEG-*sacB* Red recombinase vector, *sacB*^+^, Cm^R^, ([Derbise *et al*., 2003](#_ENREF_19))

pPD297 pACYC184, *inv*::Tn*pho*A(60), Cm^R^ (Heroven *et al*., 2004)

pTS02 pGP20, Ap^R^ (Böhme et al. 2012)

pTS03 pSC101*, promoter probe vector, -*lacZ*, Ap^R^ Tatjana Stolz

pTT1 pTS03, P*_tet_*::*lacZ,* Ap^R^ this study

a the number indicates the codon of *rovA*, in which the resistance cassette has been inserted

b the number indicates the codon of the corresponding gene fused to *lacZ*

c the number indicates the nucleotide of the corresponding gene introduced for the *lacZ* gene

d the number indicates the nucleotides deleted from the *csrC* gene

**References:**

Böhme, K., R. Steinmann, J. Kortmann, S. Seekircher, A. K. Heroven, E. Berger, F. Pisano, T. Thiermann, H. Wolf-Watz, F. Narberhaus & P. Dersch, (2012a) Concerted actions of a thermo-labile regulator and a unique intergenic RNA thermosensor control *Yersinia* virulence. *PLoS Pathogens* **8**: e1002518.

Bolin, I., I. Norlander & H. Wolf-Watz, (1982) Temperature-inducible outer membrane protein of *Yersinia pseudotuberculosis* and *Yersinia enterocolitica* is associated with the virulence plasmid. *Infection and Immunity* **37**: 506-512.

Chang, A. C. & S. N. Cohen, (1978) Construction and characterization of amplifiable multicopy DNA cloning vehicles derived from the P15A cryptic miniplasmid. *Journal of Bacteriology* **134**: 1141-1156.

Derbise, A., B. Lesic, D. Dacheux, J. M. Ghigo & E. Carniel, (2003) A rapid and simple method for inactivating chromosomal genes in *Yersinia*. *FEMS Immunol Med Microbiol* **38**: 113-116.

Herbst, K., M. Bujara, A. K. Heroven, W. Opitz, M. Weichert, A. Zimmermann & P. Dersch, (2009) Intrinsic thermal sensing controls proteolysis of *Yersinia* virulence regulator RovA. *PLoS Pathogens* **5**: e1000435.

Heroven, A., K. Bohme, M. Rohde & P. Dersch, (2008) A Csr-type regulatory system, including small non-coding RNAs, regulates the global virulence regulator RovA of *Yersinia pseudotuberculosis* through RovM. *Molecular Microbiology* **68**: 1179-1195.

Heroven, A., G. Nagel, H. J. Tran, S. Parr & P. Dersch, (2004) RovA is autoregulated and antagonizes H-NS-mediated silencing of invasin and *rovA* expression in *Yersinia pseudotuberculosis*. *Molecular Microbiology* **53**: 871-888.

Heroven, A. K. & P. Dersch, (2006) RovM, a novel LysR-type regulator of the virulence activator gene *rovA*, controls cell invasion, virulence and motility of *Yersinia pseudotuberculosis*. *Molecular Microbiology* **62**: 1469-1483.

Heroven, A. K., M. Sest, F. Pisano, M. Scheb-Wetzel, R. Steinmann, K. Bohme, J. Klein, R. Munch, D. Schomburg & P. Dersch, (2012) Crp induces switching of the CsrB and CsrC RNAs in *Yersinia pseudotuberculosis* and links nutritional status to virulence. *Frontiers in Cellular and Infection Microbiology* **2**: 158.

Kusmierek M, Hoßmann J, Witte R, Opitz W, Vollmer I, Volk M, *et al*. A bacterial secreted translocator hijacks riboregulators to control type III secretion in response to host cell contact. *PLoS Pathogens*. 2019;15: e1007813.

Manoil, C. & J. Beckwith, (1986) A genetic approach to analyzing membrane protein topology. *Science* **233**: 1403-1408.

Nagel, G., A. Lahrz & P. Dersch, (2001) Environmental control of invasin expression in *Yersinia pseudotuberculosis* is mediated by regulation of RovA, a trans­criptional activator of the SlyA/Hor family. *Molecular Microbiology* **41**: 1249-1269.

Nuss A.M., Schuster F., Heroven AK., Heine W., Pisano F., & Dersch P. (2014) A direct link between the global regulator PhoP and the Csr regulon in *Y. pseudotuberculosis* through the small regulatory RNA CsrC. *RNA Biology* 11(5): 580-93.

Studier, F. W. & B. A. Moffatt, (1986) Use of bacteriophage T7 RNA polymerase to direct selective high-level expression. *J. Mol. Biol.* **189**: 113-130.

Takeshita, S., M. Sato, M. Tabo, W. Masahashi & T. Hashimoto-Gothoh, (1987) High-copy-number and low-copy-number plasmid vectors for *lacZ* α-complemen­tation and chloramphenicol- or kanamycin resistance selection. *Gene* **61**: 63-74.
