# Supplementary material for: The Small Protein YmoA Controls the Csr System and Adjusts Expression of Virulence-Relevant Traits of Yersinia pseudotuberculosis
Source: Front Microbiol. 2021 Aug 3;12:706934. doi: 10.3389/fmicb.2021.706934 (PMC8369931; doi:10.3389/fmicb.2021.706934)
Supplement: Supplementary file 10 [file Table_2.DOCX]

**Table S2.** Oligonucleotides used in this study.

| **Number** | **sequence** | **Site^a^** |
| --- | --- | --- |
| 1 | GCGGCGGTCGACCTCTTGGCGACAGCCATC | *Sal*I |
| 2 | GGGCGCGGATCCGCTAAGCAGACTATTTCAC | *Bam*HI |
| 3 | GCGGCGGAATTCCCTTCATCCCGTGGTAGG | *Eco*RI |
| 5 | GGGCCGGTCGACCTGAGTAACTGTGCTCCTTG | *Sal*I |
| 6 | GGGCCGGTCGACCCTTAAACATAAGCCACTCATCC | *Sal*I |
| 7 | GGGCCGGTCGACCCATTACGTTCCTTAAACATAAG | *Sal*I |
| 8 | GGGCCGGTCGACCTTGTATATCCATTACGTTCC | *Sal*I |
| 9 | GGGCCGGTCGACCTTACAGCGCACTCCCTGCAC | *Sal*I |
| 10 | GCGGCGGTCGACCGATTGCGCCTGCTAGC | *Sal*I |
| 11 | GGGCGCGGATCCGCAATGGGCTTTATTCAGC | *Bam*HI |
| 12 | GGGCCGGTCGACCTTGTATATCCATTACGTTCCTT - CCTTGCATGGTGT ATCCGTACC | *Sal*I |
| 13 | GCGCGGGTCGACT*CCCTATCAGTGATAGAGATTGACATCCCTATCAGTGATAGAGATACTGAGCAC*TTGGTACGGATACACCAT | *Sal*I |
| 14 | CGGCGCGGATCCGAAAGAAGAGAAAGAAAAAAG | *Bam*HI |
| 15 | GCGGCGGTCGACCCTTCATCCCGTGGTAGG | *Sal*I |
| 16 | GGGCGCGGATCCGATTGGGCCGGAATCTAGC | *Bam*HI |
| 23 | GCGCCTGCAG*TCCCTATCAGTGATAGAGATTGACATCCCTATCAGTGATAGAGATACTGAGCAC*CCAAGCTTCTAGTTAGGAG | *Pst*I |
| 24 | GCGCGATATCCTGCACCATCGTC | *Eco*RV |
| 25 | GGTGATTTTGAACTTTTGCTTTG (Kan1) |  |
| 26 | CCAGTGTTACAACCAATTAACC (Kan2) |  |
| 27 | GTGTAGGCTGGAGCTGCTTC (Kan3) |  |
| 28 | CATATGAATATCCTCCTTAGTTCC (Kan4) |  |
| 29 | GGGCGC**GTAATACGACTCACTATAG**TTGGTACGGATACACCATGC |  |
| 30 | CCAGTGTCCTAACATCCCT |  |
| 31 | GGGCGC**GTAATACGACTCACTATAG**GCCTGGCGGCCATAGCG |  |
| 32 | GCCTGGCAGTGTCCTACTC |  |
| 35 | GCAAACTCAGCAAGTTCTGGC | forward L-PSP |
| 36 | CGCAGGGTCTGCGCCCTC | reverse L-PSP |
| 37 | GCTGGTTTGTCTGTTTCCGAC | forward *dnaK* |
| 38 | GCACAGCGGCACCAATGGC | reverse *dnaK* |
| 39 | GCAGCGTGCATCCCGTGGTTC | forward *dnaJ* |
| 40 | GGTTTAGCACCGCTACCGTGG | reverse *dnaJ* |
| 45 | CCGACGTAAAGCCGCGATAC | forward  *sopB* |
| 46 | CCTCGTTCATAAGCACTCGTC | reverse  sopB |
| 51 | GCGCGAAAGCCTGTTACGCGC | forward *grpE* |
| 52 | CGCTCCAGATTGTCAATCACTGG | reverse *grpE* |

The corresponding restriction sites are underlined, and gene deletions marked by a line. Italic letters indicate the *tet* promoter sequence, whereas the T7 promoter sequence is given in bold.
