# Supplementary material for: The Small Protein YmoA Controls the Csr System and Adjusts Expression of Virulence-Relevant Traits of Yersinia pseudotuberculosis
Source: Front Microbiol. 2021 Aug 3;12:706934. doi: 10.3389/fmicb.2021.706934 (PMC8369931; doi:10.3389/fmicb.2021.706934)
Supplement: Supplementary file 11 [file Table_3.DOCX]

**Table S3.** Primers used for the generation of deletion mutants.

| Mutant name^a^ | Primer name | Primer sequence |
| --- | --- | --- |
| YP73  YP75 | *ymoA*::kan^a^ for | GGTTAATTGGTTGTAACACTGGCTGCTTAGCGCT GGTTAAGACACACAACGTTGAGCCGATAATCTCT ATCG |
|  | *ymoA*::kan^a^ rev | GCAAAGCAAAAGTTCAAAATCACCGGTTTTTCT TCTCGATATACAAATTAATATTGGTGGAACTAT CCC |
|  | *ymoA* for | CGATAGACAGCTGTATTTATATG |
|  | *ymoA* rev | CCTGTATTATCACTTTCCTGC |

^a^ The *Y. pseudotuberculosis* mutants were constructed by adding a kanamycin resistance cassette (Kan). Underlined bases correspond to the homologous nucleotides of the resistance gene. Rev: reverse primer; for: forward primer.
